# Next-Generation Sequence Analysis of Cancer Xenograft Models

**DOI:** 10.1371/journal.pone.0074432

**Published:** 2013-09-26

**Authors:** Fernando J. Rossello, Richard W. Tothill, Kara Britt, Kieren D. Marini, Jeanette Falzon, David M. Thomas, Craig D. Peacock, Luigi Marchionni, Jason Li, Samara Bennett, Erwin Tantoso, Tracey Brown, Philip Chan, Luciano G. Martelotto, D. Neil Watkins

**Affiliations:** 1 Monash Institute of Medical Research, Monash University, Clayton, Victoria, Australia; 2 Department of Pathology, University of Melbourne, Parkville, Victoria, Australia; 3 Molecular Genomics Core Facility, Peter MacCallum Cancer Centre, East Melbourne, Victoria, Australia; 4 Department of Anatomy and Developmental Biology, Monash University, Clayton, Victoria, Australia; 5 Sir Peter MacCallum Department of Oncology, University of Melbourne, Parkville, Victoria, Australia; 6 Department of Biochemistry and Molecular Biology, Monash University, Clayton, Victoria, Australia; 7 Department of Cancer Medicine, Peter MacCallum Cancer Centre, East Melbourne, Victoria, Australia; 8 Department of Oncology, Sidney Kimmel Comprehensive Cancer Centre, Johns Hopkins University School of Medicine, Baltimore, Maryland, United States of America; 9 Bioinformatics Core Facility, Peter MacCallum Cancer Centre, East Melbourne, Victoria, Australia; 10 Department of Mechanical Engineering, University of Melbourne, Parkville, Victoria, Australia; 11 Partek SG Private Limited, Singapore, Republic of Singapore; 12 Monash eResearch Centre, Monash University, Clayton, Victoria, Australia; 13 Department of Pathology, Memorial Sloan-Kettering Cancer Center, New York, New York, United States of America; University of North Carolina School of Medicine, United States of America

## Abstract

Next-generation sequencing (NGS) studies in cancer are limited by the amount, quality and purity of tissue samples. In this situation, primary xenografts have proven useful preclinical models. However, the presence of mouse-derived stromal cells represents a technical challenge to their use in NGS studies. We examined this problem in an established primary xenograft model of small cell lung cancer (SCLC), a malignancy often diagnosed from small biopsy or needle aspirate samples. Using an *in silico* strategy that assign reads according to species-of-origin, we prospectively compared NGS data from primary xenograft models with matched cell lines and with published datasets. We show here that low-coverage whole-genome analysis demonstrated remarkable concordance between published genome data and internal controls, despite the presence of mouse genomic DNA. Exome capture sequencing revealed that this enrichment procedure was highly species-specific, with less than 4% of reads aligning to the mouse genome. Human-specific expression profiling with RNA-Seq replicated array-based gene expression experiments, whereas mouse-specific transcript profiles correlated with published datasets from human cancer stroma. We conclude that primary xenografts represent a useful platform for complex NGS analysis in cancer research for tumours with limited sample resources, or those with prominent stromal cell populations.

## Introduction

Although the application of NGS technology to cancer research has led to dramatic advances in the understanding of the genomic basis of these diseases, the depth and complexity of sequencing data is negatively correlated to the amount and quality of tumour specimen used for analysis [1]. In addition, many common tumours, such as pancreatic cancer, are characterized by extensive infiltration of stromal elements, thereby reducing the detection threshold for rare, cancer specific variants [2]. As a result, common cancers diagnosed by small biopsies are vastly underrepresented in NGS studies, which rely predominantly on surgically-resected tissue samples.

One approach to overcome this problem is the use of primary xenograft models, in which small tissue samples can be directly engrafted, expanded and passaged in immunodeficient mice without exposure to conventional tissue culture conditions [3]. Although tumour cells are maintained in immunodeficient mice, we [4], and others [5]–[7], have shown that they retain important characteristics of the primary tumour that, importantly, are irreversibly lost in cell culture [2], [4]. Moreover, despite the fact that the stromal component is mouse-derived, primary xenograft models have been successfully used for the preclinical investigation of a variety of cell autonomous and stromal derived signaling systems of therapeutic relevance to cancer [7].

Based on these data, primary xenografts could represent a useful platform for NGS analysis when cancer tissue is limiting. Ding *et al.* [8], in a study that aimed to identify somatic mutations and structural variants of basal-like breast cancer, estimated by pathology techniques the tumor composition to then calculate and adjust the tumour read number. Based on the pathology estimates, the authors use a deterministic correction of contamination of tumour by normal read counts, which affects the mutant allele frequency, and applied it to the primary tumour and metastasis samples only. It was assumed that due to the low mapping rate of host-specific reads to the graft genome, no read depth correction was required to the xenograft sample.

In our view, the presence of contaminating mouse DNA and RNA affects the sensitivity and specificity of NGS analysis in these tumour models which should not be based on cellularity estimates, but should be accurately and systematically addressed. Additionally, since most current NGS techniques use shotgun-sequencing methodology, resolution of any potential artifact could be performed *post-hoc* during bioinformatic analyses, which unequivocally identify species-of-origin reads. This issue has been previously discussed for ultra high-throughput cDNA sequencing (RNA-Seq) by Conway *et al.* [9] and Raskatov *et al.* [10], who found variable amounts of host-derived sequencing reads. Here, we prospectively analyzed the capacity of an *in silico* workflow designed to definitively assign species-of-origin to NGS reads in several previously characterized primary and cell line-derived xenograft models of SCLC, and compared these results with published datasets.

## Materials and Methods

### Ethics Statement

All experiments involving animals were approved in advance by an Animal Ethics Committee at Monash University and were carried out in accordance with “Australian Code of Practice for the Care and Use of Animals for Scientific Purposes.”

### Cells

The SCLC primary xenograft lines LX22, LX33 and LX36 were passaged as previously described [4]. In brief, resected tissues from chemo-naïve SCLC patients were used to generate primary xenografts samples. Tumour samples were finely chopped with sterile razor blades, triturated in 1 x PBS, filtered through a 60 µm mesh filter, centrifuged and resuspended in 500 µL of Matrigel (BD Biosciences) at 4 °C. Processed cells were then injected sub-cutaneously in the flanks of non-obese diabetic/severe combined immunodeficient mice. Once the P0 tumours reached a diameter of 1 cm, the mouse was sacrificed and the resected tumour was divided into sections for snap freezing or serial passage. Xenograft tumours were prepared for serial passages *in vivo* as described above and cells were injected into the flanks of athymic nude mice in Matrigel. Passaged and snap frozen tumours samples were routinely characterised for histopathologic and immunohistochemical features of the parent tumour [4].

Authenticated NCI-H209 cell line was purchased from ATCC, re-derived from a single cell clone using the single cell cloning by serial dilution (Corning, Tewksbury, MA, USA) and then cultured *in vitro* and in vivo as described in Watkins *et al.* [11]. DNA from samples was extracted using DNAeasy Tissue and Blood Kit (Qiagen, Santa Clara, CA, USA) according to manufacturer’s instructions. RNA was purified using miRNeasy Mini Kit using QIAzol (Qiagen, Santa Clara, CA, USA) following manufacturer’s instructions.

### Preparation of Sequencing Libraries

Exome and low-coverage whole-genome DNA re-sequencing: Target DNA (3ug) was firstly sheared using a focal acoustic device (Covaris, Woburn, MA, USA). DNA fragment libraries for exome re-sequencing and low-coverage whole-genome sequencing were constructed from sheared DNA by sequential steps of end-repair, A-tailing and ligation of indexed lllumina compatible adapter sequences (TruSeq DNA, Illumina, San Diego, CA, USA). For exome re-sequencing, PCR amplified fragment libraries were enriched for exonic DNA by long oligonucleotide hybridisation capture according to the manufacturer’s protocol (SeqCap EZ Exome Library v3.0, Roche Nimblegen, Madison, WI, USA). For low-coverage whole-genome, PCR-amplified libraries were size selected to capture DNA of 500–700nt length, using an automated electrophoresis platform (Pippen Prep, Sage Science Inc., Beverly, MA, USA). All sequencing libraries were quantified using real-time PCR against a library of known concentration and then processed for cluster generation and sequencing according to standard protocols (HiSeq 2000, Illumina, San Diego, CA, USA).

#### RNA-Seq

total RNA was checked for quality and yield by automated microfluidic electrophoresis (Bioanalyzer 2100, Agilent Technologies, Santa Clara, CA, USA) and spectrophotometer (NanoDrop, Thermo Scientific, Wilmington, DE, USA). Non-directional RNA-Seq libraries were created according to the manufacturers protocol (Truseq RNA-Seq Library Prep Kit v2, Illumina, San Diego, CA, USA). Briefly this method involved sequential steps of mRNA enrichment from 3ug total RNA, RNA fragmentation by heating in the presence of divalent cations, a randomly primed reverse transcription and second-strand cDNA synthesis followed by preparation of DNA fragment libraries using Illumina compatible adapters and PCR amplification as previously described for DNA libraries.

All samples were assessed separately for overall read quality using FASTQC (http://www.bioinformatics.bbsrc.ac.uk/projects/fastqc) and low quality reads were filtered and were hard trimmed using Trimmomatic (average minimum Phred score, 6 consecutive bases, of 20 and a minimum read length of 50nt, Table S1) [12].

Raw deep sequencing datasets are publicly available in the National Centre of Biotechnology Information Short Read Archive (Accession number SRA082685).

### Strategy to isolate and identify species-of-origin NGS reads

The proposed strategy resembles that described by Conway *et al.* [9], but differs in several important aspects. First, a primary alignment to the graft genome, in this case the human genome, is performed, where reads are divided into graft-mapped and graft-unmapped reads; second, both graft-mapped and graft-unmapped read-sets are realigned to the host genome, in this case the mouse genome, to further identify common graft-host and host-specific reads respectively; lastly, common graft-host reads are filtered from the read set obtained in the primary alignment to obtain graft-specific reads. In this study, the identification and classification processes were performed *via* collecting and comparing the read ids of the host/graft alignments, producing reads in FASTQ format. As a result, identified graft-specific reads were re-aligned to the graft genome.

**Figure 1:**
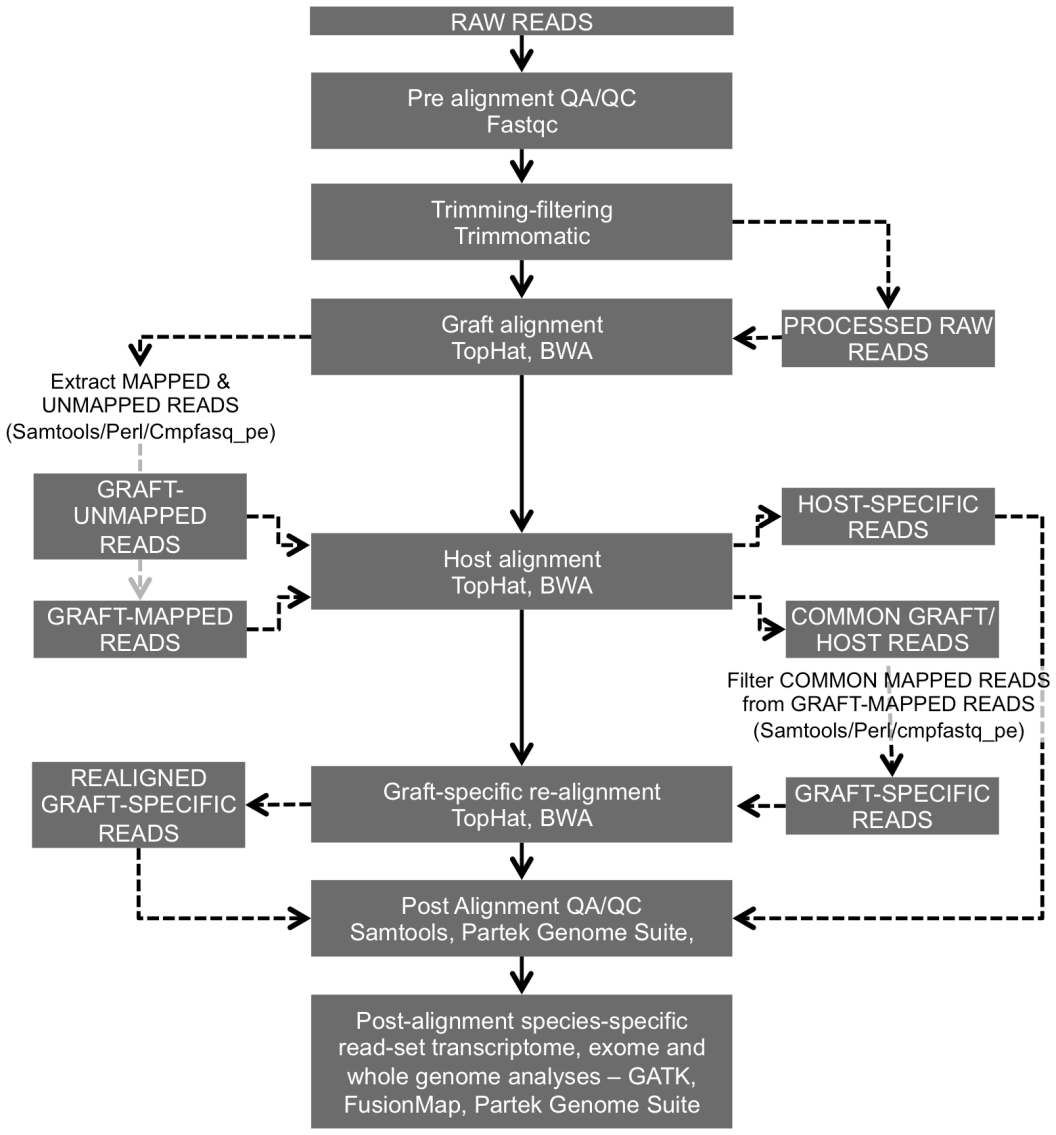
Overview of the steps followed to identify and isolate common and species-specific sequence reads, including gene identification and pathway analysis. The software components utilized in each step are also specified. Solid lines represent the principal analytical path followed and dashed lines represent auxiliary steps.

Subsequent alignments produced three separate aligned datasets, *i. e.*, reads that could only be mapped to the human genome, reads that were exclusively mapped to the mouse genome and reads that mapped to both genomes. In addition to analysing RNA-Seq read sets, we further verify this strategy for low-coverage whole-genome and exome-capture sequencing experiments. A complete overview describing all the steps included in the proposed strategy is shown in Figure 1. For each alignment, mapped and unmapped reads contained in SAM/BAM formatted files [13] were filtered based on their bitwise flag status using Samtools [13], a customised Perl script that collected unique read identities from the aligned/unaligned SAM formatted files and filtered them from the raw fastq files, [Simon Andrews, 2010, Seqanswers.com [14]. Available at: http://seqanswers.com/forums/showpost.php?p=25302&postcount=3] and the cmpfastq_pe software, that compared raw pair-end fastq files and reported common and unique reads (http://compbio.brc.iop.kcl.ac.uk/software/cmpfastq_pe.php).

Mapping scores were used to assess the mapping quality of the processed samples and to further discard multiple-hit reads. As a general rule, it was assumed that a higher mapping quality means a more “unique” aligned read and for most of the samples, a high percentage of the read-pairs had a mapping quality above 20 (Table S2).

### Transcriptome analysis

Whole transcriptome analysis of three SCLC primary xenografts was performed through RNA-Seq using the GAIIX and HiSeq 2000 sequencing platforms (Illumina**,** San Diego, CA, USA). The experiment was paired-end with 100nt read length (300nt average insert size). The targeted minimum number of reads per sample was 40 million reads (Table S1).

In order to identify and unequivocally separate graft (human) and host (mouse) reads, processed sample reads were sequentially aligned to both graft [complete hg19 human genome (UCSC version, February 2009)] and host [complete mm9 mouse genome (UCSC version, July 2007)] genomes using Bowtie-TopHat [version 2.0.4, segment length 29nt, 1 mismatch in segment permitted, for maximum sensitivity, coverage search performed [15], [16]. No de-duplication was performed for post-assembly RNA-Seq analysis.

mRNA quantification for all annotated genes from the human genome was performed using Partek**®** software (Partek Inc. (1993) Partek® Genomics Suite™). Reads were normalized using the reads per kilobase of exon model per million mapped reads method [17].

A human-specific primary xenograft microarray expression data-set (GSE15240) [4] was retrieved from the National Center for Biotechnology Information (NCBI) Gene Expression Omnibus (GEO) repository [18].

To compare the mouse-specific reads to previously published cancer stromal gene signatures, a breast cancer associated fibroblasts dataset [19] was retrieved from the GEO repository (GSE10797).[18]

For all microarray analysis, gene probes were normalized using quantile normalization (log base 2 and median polish for probeset transformation and summarization respectively) and background correction was performed using the robust multi-array average method (RMA) [20].

Comparison of microarray and RNA-Seq gene expression results was performed using linear correlation (Spearman’s r) between the log base 2 of the quantified gene arbitrary intensity units and the log base 2 RPKM as described in Mortazavi *et al* [17].

### Exome resequencing analysis

Whole-exome analysis of samples obtained from peripheral blood, NCI-H209 cell line and its derivative xenograft was performed through whole exome ultra-high throughput sequence using the HiSeq 2000 sequencing platform (llumina**,** San Diego, CA, USA). The experiment was paired-end with 101nt read length (200bp insert size). The average targeted depth of coverage was set to 50x (see Table S1 for total number of reads sequenced).

Processed sample reads were sequentially aligned to both graft [complete hg19 human genome (UCSC version, February 2009)] and host [complete mm9 mouse genome (UCSC version, July 2007)] genomes using the Burrows-Wheeler Alignment tool [(BWA), bwa aln algorithm used, seed length of 22nt; maximum edit distance in the seed of 0 [21].

Single nucleotide variants (SNVs) discovery was performed using a set of tools included in Picard (http://picard.sourceforge.net) and GATK [22], [23]. First, duplicate reads were removed from the realigned BAM files using the MarkDuplicates command from Picard (http://picard.sourceforge.net). Estimated duplication levels are described in Table S3. Subsequently, de-duplicated BAM files were locally realigned around novel and known indels using the RealignerTargetCreator and the IndelRealigner walkers from GATK [23]. Lastly, base quality scores were recalibrated using the CountCovariates and TableRecalibration walkers from GATK [23]. This procedure was performed for each of the three samples analysed.

Raw SNP calls were performed using the UnifiedGenotyper walker from GATK [23] with a minimum base quality Phred score of 20, a call confidence threshold of 50 (Phred-scaled) and an emmition confidence threshold of 10 (Phred-scaled). Raw called SNPs were filtered using the VariantFiltration walker with the following parameters: SNP cluster size  =  10; Coverage: ≥ 5; Qual: ≥ 50; Strand bias: Fisher’s exact test, ≥ 60. Sample-specific novel SNPs, *i. e.*, those not present in the Database of Single Nucleotide Polymorphisms (dbSNP) (Bethesda (MD): National Center for Biotechnology Information, National Library of Medicine. (dbSNP 137: 137; http://www.ncbi.nlm.nih.gov/SNP/), were annotated and its effect predicted using SnpEff [24] and the variantAnnotator walker from GATK [23].

Genome visualization was performed using the Integrative Genome Browser (IGV) [25], [26]. Multispecies local alignment tracks were retrieved from IGV data server.

### Whole-genome analysis

A low-coverage whole-genome sequencing of samples obtained from peripheral blood, H209 cell line and its derived primary xenograft was performed through shotgun whole genome ultra-high throughput sequence using the HiSeq 2000 sequencing platform (llumina**,** San Diego, CA, USA). The experiment was paired-end with 101nt read length (200bp insert size). The average targeted depth of coverage was set to 4x (see Table S1 for total number of reads sequenced).

Processed sample reads were sequentially aligned to both graft [complete hg19 human genome (UCSC version, February 2009)] and host [complete mm9 mouse genome (UCSC version, July 2007)] genomes using the Burrows-Wheeler Alignment tool [(BWA), bwa aln algorithm used, seed length of 22nt; maximum edit distance in the seed of 0 [21]. Estimated duplication levels were found to be marginal and are described in Table S3.

Intra- and inter-chromosomal rearrangements discovery of the identified human specific reads was performed using FusionMap [span and split read count threshold of 3 and split minimum anchor of 4 reads [27]. Detected fusions were plotted against a circular representation of the human genome (Circos plot) using Circos [28].

Copy number variations (CNV) and allelic content in genomic regions were detected using Control-Freec [29]. The peripheral blood sample was used as a baseline control. Circos plots of the detected CNV were built using Circos [28].

## Results

**Figure 2:**
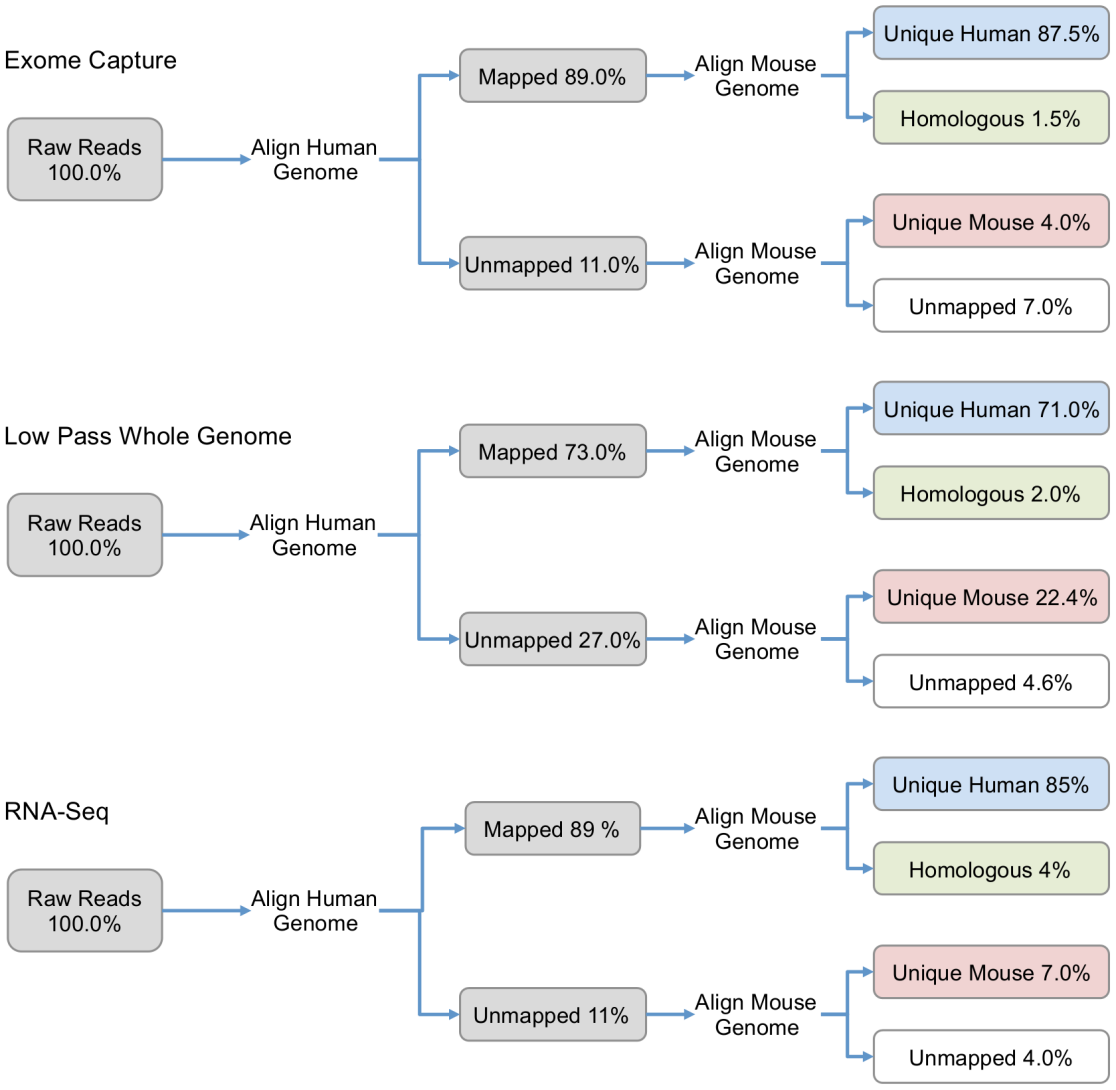
Summary of the results produced by the proposed strategy to isolate and identify species-specific NGS reads in human xenografts. For each read category, the proportion (%) of the total number of reads is specified.

As shown in Figure 2, the assessed NGS strategies revealed different proportions of host-specific reads. Exome capture and RNA-Seq produced the lowest proportion of mouse specific reads, ranging from 4% to 7%. In contrast, shotgun whole genome sequencing produced the highest number of reads that uniquely aligned to the mouse genome, which corresponded to 20% of the total number of reads (Figure 2). The homologous number of reads, *i.e.*, those reads that aligned to both the human and the mouse genome, was found to be similar for all methods, ranging from 4% (RNA-Seq) to 1.5% (Exome-capture). A complete summary of the alignments performed is described in Table S2.

### Whole-genome analysis

As expected, the sequence depth of coverage of the samples subjected to low-coverage whole-genome sequencing was above 3 times for all analysed samples (Table S3 A). However, the depth of coverage of the xenograft sample was severely affected by mouse contamination and produced the lowest value of the 3 samples both for mean depth of coverage (3.3 times) and percentage of reads covered at least 3 times (Table S3 A).

**Figure 3:**
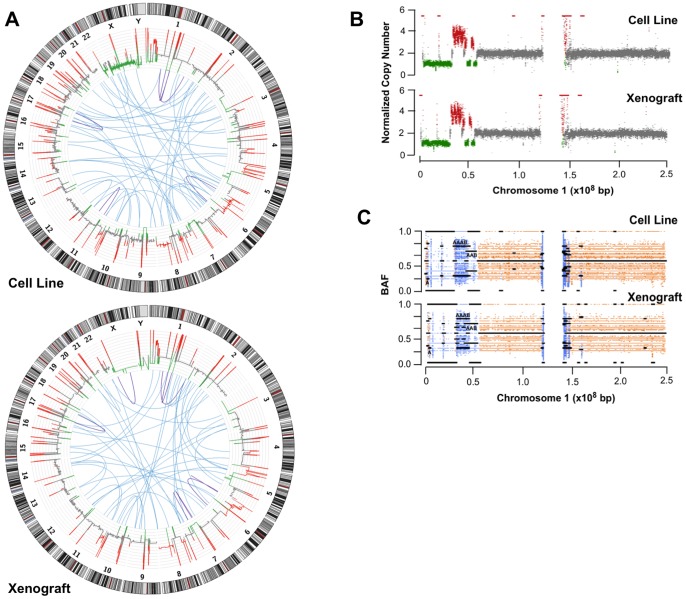
Copy number variations, inter and intra-chromosomal rearrangements and B allele frequencies of NCI-H209 cell line and a xenograft tumour derived from it. (A) Circos plot representing copy number variations, inter and intra-chromosomal rearrangements of NCI-H209 cell line and a xenograft tumour derived from it. Copy number variations (red, gain; green, loss) were calculated based on coverage using the correspondent peripheral blood as control. Inter and intra-chromosomal rearrangements are represented in blue (inter-chromosomal) and dark blue (intra-chromosomal). (B, C) Detailed profile of copy number variations and B-allele frequencies of chromosome 1 from the analysed cell line and xenograft. As described above, the correspondent peripheral blood was used as control for both type of analysis. Copy number profiles are shown in red (gain), green (loss) and grey (no change). LOH are shown light blue.

Copy number variation analysis of both the cell line and xenograft samples produced highly similar results when the peripheral blood sample was used as control (Figure 3 A). A total of 578 and 470 somatically acquired copy number alterations were observed for the cell line and xenograft samples respectively. These differences were mainly due to the subtle differences in the depth of coverage of the genomic regions assessed and most of them correspond to focal copy number gains or losses in the middle of diploid regions (Figure 3 B). As observed in Figure S1, both the cell line (Figure S1 A) and xenograft (Figure S1 B) samples produced highly similar CNV profiles for all the analysed chromosomes. A detailed CNV profile of both samples can be found in Datasets S1 and S2. A similar pattern was observed for *beta* allele frequency profiles for both sample types (Figure 3 C).

Comparable results could be observed for intra- and inter-chromosomal rearrangements (Figure 3 A), where over 70 rearrangements for both samples were detected. An example of inter-chromosomal rearrangements was found between *BAGE4*, a candidate gene encoding tumour antigens, and *MLL3*, a member of the myeloid/lymphoid or mixed-lineage leukemia (MLL) family. A complete list of the intra- and inter-chromosomal rearrangements common to both cell line and the xenograft samples can be found in Datasets S3.

The data presented above supports our hypothesis that a thorough CNV and structural variant analysis can be performed when both the cell line and xenograft samples were used. We found that when correctly accounting for mouse-specific contamination, the results obtained using uncontaminated cell lines can be accurately reproduced using xenograft samples, with the additional benefits of the usage of an *in vivo* model.

### Exome sequencing analysis

A mean sequence depth of coverage in the targeted captured regions in all samples of over 100 times was achieved, with more than 80% of the bases covered at least 30 times (Table S3 B). In the cell line and the xenograft samples, 68.5 and 74.7 percent of the targeted exome regions were covered at least 50 times, with a mean sequence depth of coverage of 109 and 136 times respectively. Sequence analysis across all three samples (*i. e.*, peripheral blood, cell line and xenograft) detected a total of 53,186 (52,429 known and 757 novel) SNPs. Those variants that were found in the peripheral blood were considered of germline origin, and were no further processed for tertiary analysis.

**Figure 4:**
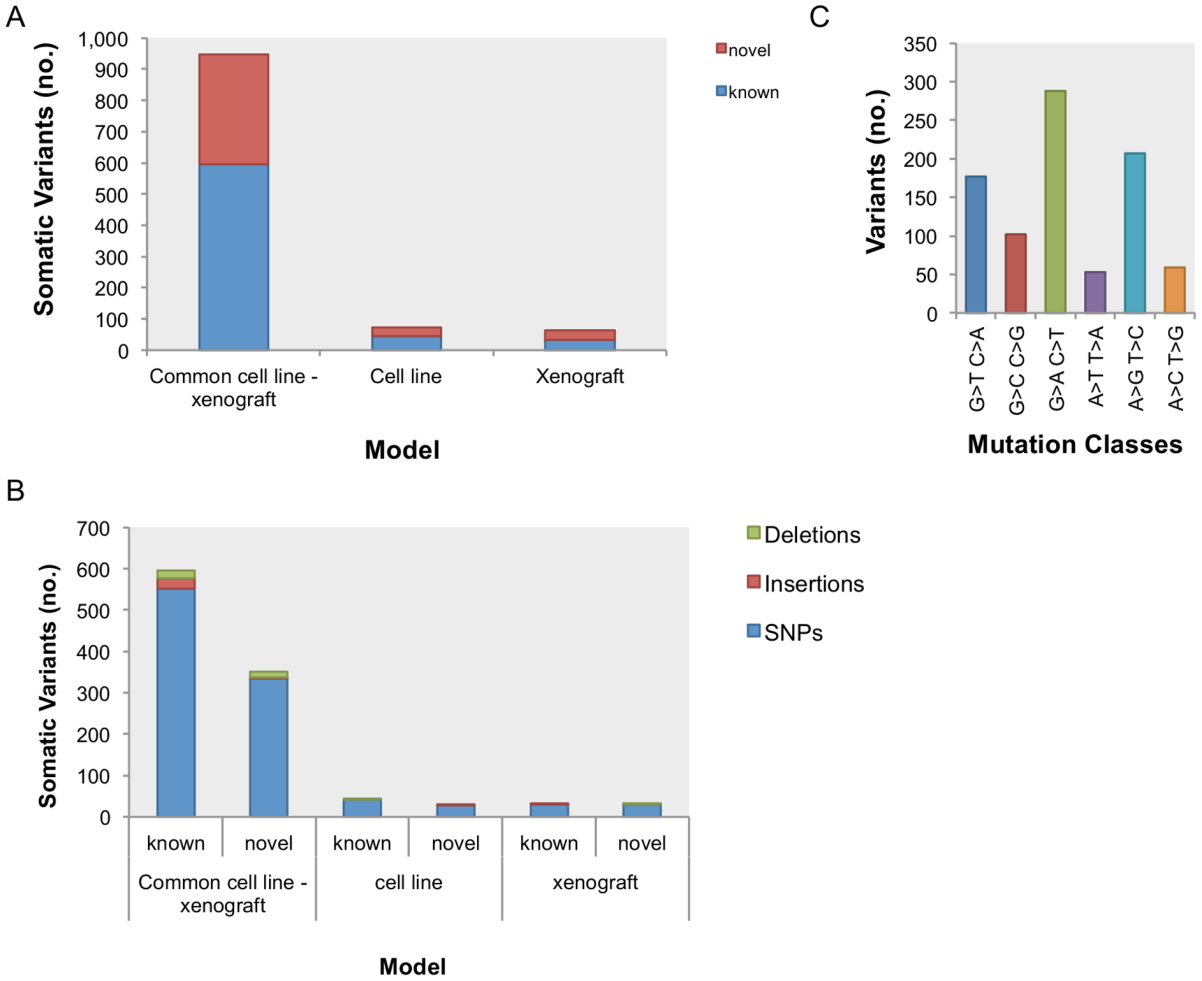
Somatic variants profile of the NCI-H209 cell line and a xenograft tumour derived from it. Number of known and novel variants (**A**) and variant types (**B**) found to be common to both the cell line and xenograft and those detected only in the cell line and xenograft. (**C**) Quantification of the six possible mutation classes.

A total of 946 somatic variants, 351 of these novel, were common to both the cell line and xenograft samples (Figure 4 A). Of these, 886 were single base substitutions, 28 were insertions and 32 were deletions (Figure 4 B). A complete list of the somatic mutations detected is described in Datasets S4. Mutation class analysis showed G>A/C>T transitions were the most common (33%) followed by A>G/T>C transitions (23%) and G>T/C>A transversions (20%) (Figure 4 C). Overall, this pattern was similar to that reported by Pleasance *et al* [30].The previously described TP53 splice acceptor disrupt and RB1 C706F point mutation, characteristic of SCLC, [30], were detected both in the cell line and xenograft samples.

**Figure 5:**
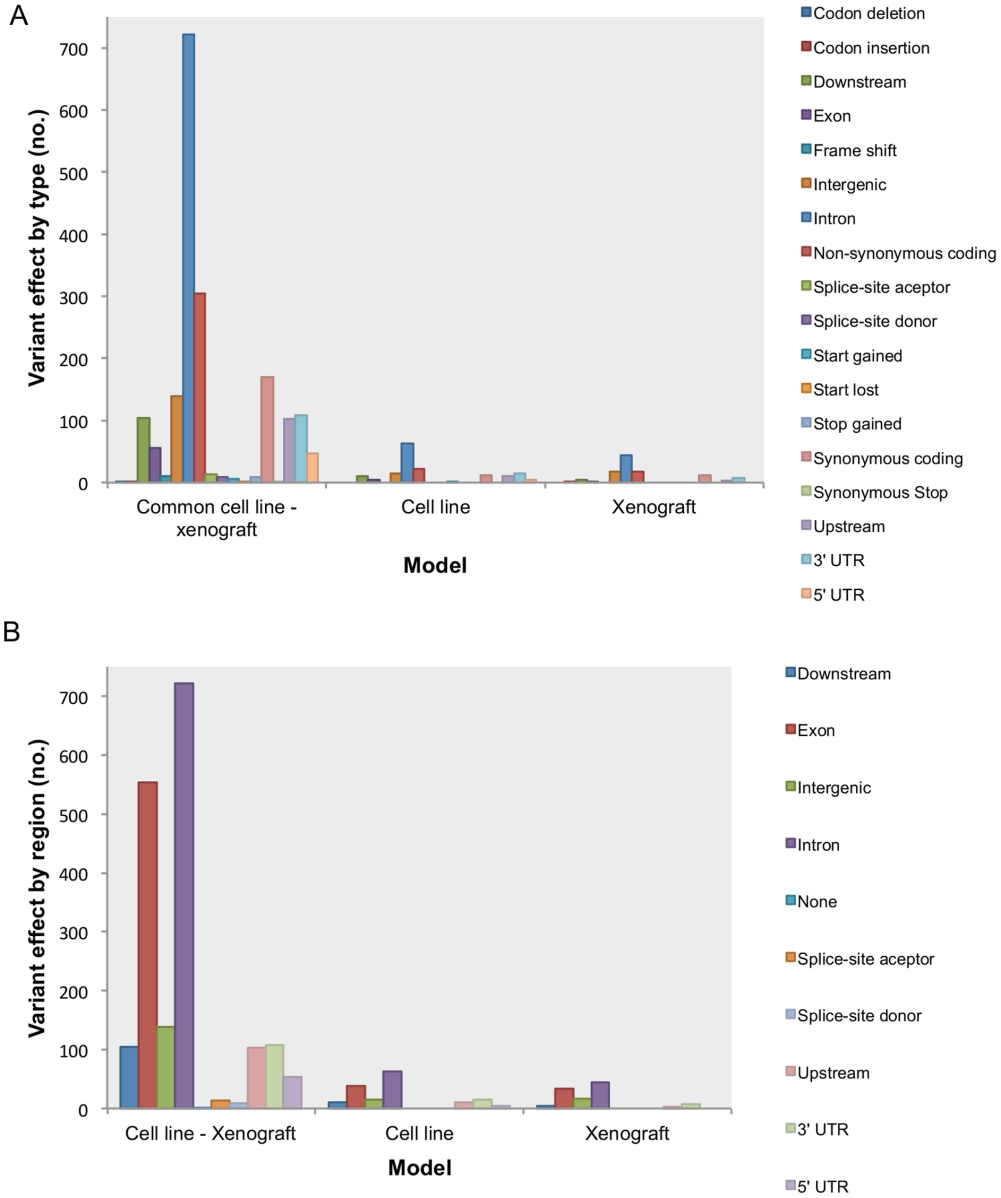
Variants classification by type of predicted effect (A) and genomic region affected (B).

For the 946 variants common to both cell line and xenograft, the SnpEff effect predictor reported a total of 1806 (Figure 5 A & B). For the purpose of this analysis, we reported the effect for all possible gene transcripts, thus the total number of reported variants differs from the total number of effects found. The most represented effects categories, when classified by type, were those corresponding to introns (721), non-synonymous coding (305) and synonymous coding (170) (Figure 5 A). When the variant effects were classified by region, intron and exon regions, as expected, were the most significantly represented (Figure 5 B). A description of moderate and high impact SNPs predicted effects for the first affected transcript is described in Datasets S5.

Sixty-four somatic variants unique to the xenograft were identified (Figure 4 B). Of these, only 15 were non-synonymous coding variants. In all cases, the variants were heterozygous, and SnpEff predicted a moderate effect on protein function (Table S4 A). These variants affected gene transcripts of the following genes: *ESPN, KAZN, APEH, MUC20, MUC17, AQP7, ZNF808* and *LUZP4*. In order to identify the cause of these discrepancies between the variants detected in the cell line and the xenograft samples, the genomic regions surrounding the variants detected were examined. In order to exclude the possibility that these variants arose from contaminating mouse sequence, we performed the following analysis. First, we isolated the sequencing reads adjacent to the region of interest within a range of 1,000bp (See Figure S2 for detailed examples). Pairwise local alignments of these regions between the human and mouse genomes showed that a global alignment could not have been possible between the analysed sequencing reads and the mouse genome (Figure S2). Next, we attempted to align these reads to the mouse genome. No alignments were produced. These data show that the coding-region variants unique to the xenograft were of human origin.

Since genetic heterogeneity is now considered a cardinal feature of many cancer types [31]–[33], we wondered whether these xenograft-specific variants could be detected in the original cell line dataset. Detailed inspection of the sequencing reads and sequence depth-of-coverage of relevant regions revealed that the great majority (9 out of 15) of these variants were detectable, but were below the allele frequency threshold of 0.2 (Figure S3 & Table S4 A). For variants not detected in the cell line, either the sequence depth of coverage was below 10 times or the alternative allele nucleotide was not observed (Table S4 A). These data support the conclusion that the variants unique to the xenograft arose as a result of clonal expansion from a heterogeneous cell line population, or new variants arising from spontaneous background mutations.

A further 74 variants were identified in the cell line, but not in the xenograft sample (Figure 4 B). Of these, 9 (*RHOA, MUC17, TRIM22, UNC93B1, MAML2, HIF1A, FAM18B2 and GPR64*) resulted in non-synonymous coding region changes with a predicted moderate impact on protein function (Table S4 B). All of these discrepant variants were found to be heterozygous (Table S4 B). A comparison of the sequencing reads and sequence depth of coverage of these regions revealed similar coverage in both cell line and xenograft sample (Table S4 B & Figure S4). Using a similar approach to that taken for the xenograft-specific variants, we determined that in all but one case, the cell line-specific variant could be readily detected in the xenograft, but once again were below the same allele frequency threshold. Since these reads were identified in a pure human cell line population, we conclude that cells containing these discrepant variants are represented at lower frequency in the xenograft, rather than as a result of mouse contamination or variation in sequencing depth.

The number of discordant variants detected for each sample – 64 xenograft specific *versus* 74 cell line specific variants – may have biased the known-to-novel ratio observed in the xenograft (Figure 4 B). This sample ratio is close to 1∶1, higher than the observed for the cell line specific and common cell line - xenograft variants which is below 1 (Figure 4 B).

The data set from the xenograft sample produced the highest mean sequence depth of coverage and 75% of the sequenced bases were covered at least 50 times. The great majority of somatic variants were detected in both cell line and xenograft, whereas variants that were uniquely detected to either in the cell line or the xenograft represented a minor proportion with no significant effect on translation of mRNA splicing. Taken together, these data show that exome-capture sequencing in xenograft models yields highly accurate and reproducible detection of significant coding-region variants.

### Transcriptome analysis

**Figure 6:**
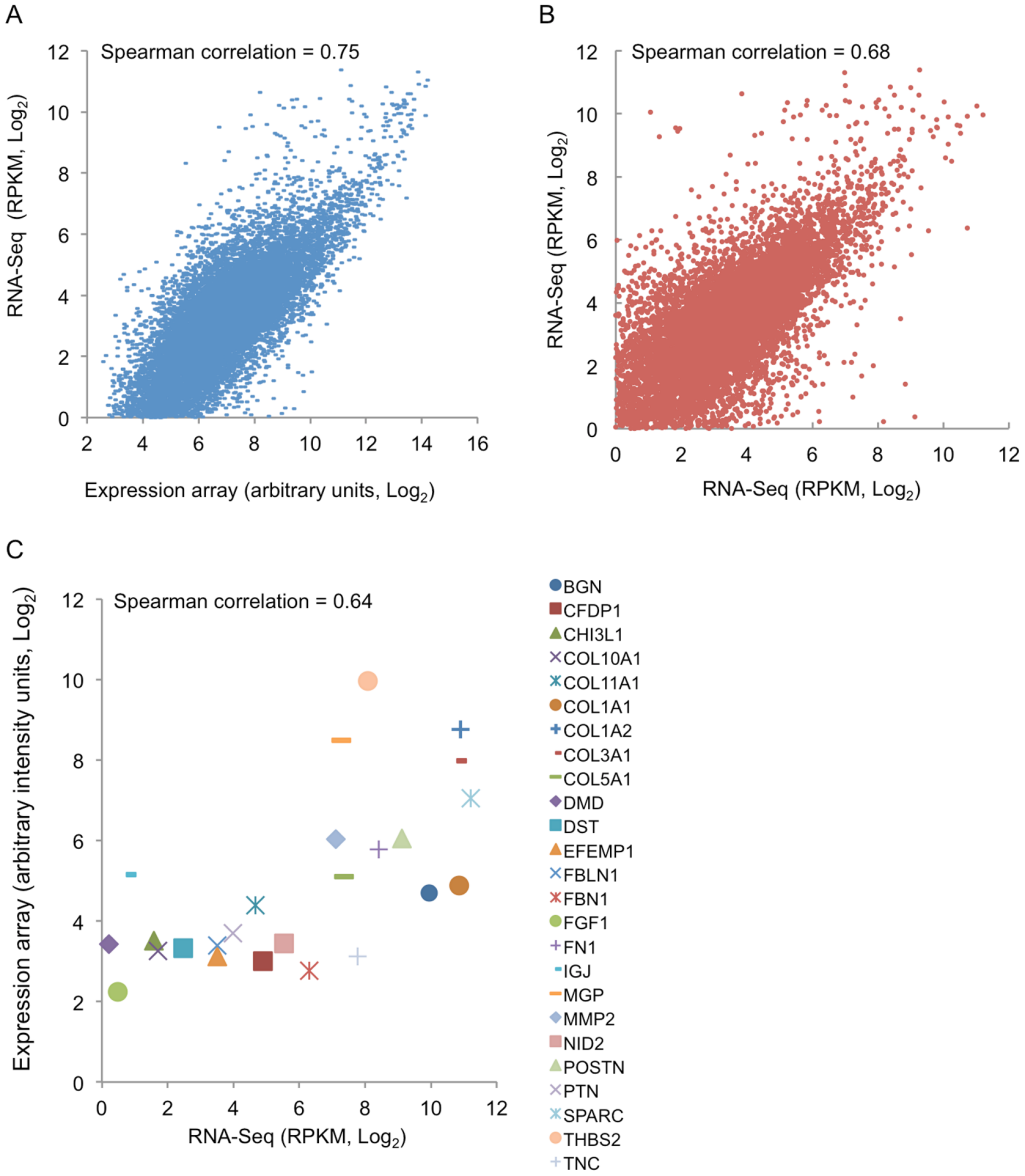
Comprehensive correlation analysis between the RNA-Seq and Affymetrix expression array platforms. (**A**) Comparison of gene expression detected by RNA-Seq and Affymetrix expression array platforms for identical SCLC samples (mean, n = 3, P<0.01). (**B**) Comparison of the gene expression between SCLC primary tumours [34] (Y axis, mean, n = 15) and primary xenografts (X axis, mean, n = 3) (P<0.01). (**C**) Comparison of gene expression detected by Affymetrix array of micro-dissected human cancer stroma [19] (Y axis, mean, n = 28) and mouse-specific RNA-Seq expression data in the SCLC xenograft models (X axis, mean, n = 3) (P<0.01).

Human-specific transcriptome analysis of three SCLC primary xenograft models (LX22, LX33 and LX36) showed a strong correlation (Spearman correlation  =  0.75, P<0.001) with a previously published gene-expression array data set in the same tumor models using human-specific cDNA probesets [4] (Figure 6 A), thus independently validating our species-specific strategy.

A gene expression correlation analysis between a recently published SCLC primary tumors RNA-Seq experiment [34] and the human-specific RNA-Seq reads of SCLC primary xenograft models, showed positive correlation between both datasets (Spearman correlation  =  0.68, P <0.001) (Figure 6 B). Isolated mouse-specific reads from the RNA-Seq experiment were compared with a laser micro-dissected human breast cancer stroma-specific gene expression array dataset [19]. As shown in Figure 6 C, a positive correlation between mouse-specific RNA-Seq expression data and the stroma-specific cancer gene signature, determined by the expression array analysis of laser micro-dissected human breast cancer tissue, was observed. As previously reported [19], genes highly expressed in cancer stroma compared to normal stroma, such as *BGN*, *COL1A1*, *COL1A2*, *COL5A1*, *FN1*, *NID2*, *COL10A*, *COL11A1*, *COL3A1*, *MMP2*, *POSTN*, *SPARC*, *DST* and *THBS2*, produced high number of RPKM and positively correlated with the gene expression array data (Figure 6 C). At the same time, genes found to be down-regulated for the same comparison, namely *FGF1*, *IGJ*, *PTN*, *MGP*, *CHI3L1*, *DMD*, *MMP7* and *EFEMP1*, were found to have low expression levels (Figure 6 C). Expression levels of *FBLN1*, *FBN1*, *CFDP1* and *NID2* were found to be low, in contrast to what reported previously [19].

The analyses described above convey and support two main hypotheses. First, that there was a high cross-platform correlation, microarray *versus* RNA-Seq, when expression was analyzed in a previously described model, *i. e.*, a primary xenograft model of SCLC [4]. Secondly, the positive correlation found between the previously published SCLC primary tumours [34] and the human-specific reads plus the positive correlation between breast cancer stroma-specific gene expression array dataset [19] and the mouse-specific reads, verifies the main hypothesis of this work where host and graft reads can be unequivocally identified and separately analyzed.

## Discussion

To date, complex NGS analyses of cancer have relied on conventional cell line models, or fresh-frozen surgical specimens. Although cell lines provide high-quality, tumour-specific RNA and DNA, the process of adapting to adherent, serum-dependent culture conditions results in irreversible transcriptional, epigenetic and genomic changes [4]–[7], which can limit the interpretation of NGS studies. Suitable freshly isolated surgical material is limited by stromal contamination and tissue quality [1], and the fact that resected cancers become over-represented in NGS studies. In addition, although complex mathematical modeling can be used to correct for sample purity and allele frequency in exome sequencing [2], [35], complex tissue samples remain a major challenge for whole-genome analysis. Our results also suggest that much larger and more complex stroma-specific gene expression studies can be undertaken to further validate primary xenograft models in tumours such as SCLC.

Using an *in-silico* workflow based on short-read aligners, such as Tophat-Bowtie and BWA, we have shown that xenograft models can be used for highly accurate, sensitive and specific NGS at a whole genome, exome and transcriptome level. Despite significant mouse contamination in both DNA and RNA analysis, we were able to derive highly accurate sequence data that was internally consistent when comparing xenografts to matched cell lines, and also replicated NGS and array-based analyses of identical cell and xenograft lines generated independently.

Conway *et al.* [9] recently described a classification technique called Xenome. This tool allows a pre-processing and further classification of high throughput sequencing reads using a k-mer decomposition of the host and graft reference genome sequences. Once processed, reads are classified into four categories: reads originated from the host tissue, reads originated from the graft, reads that could have been originated from both type of tissues and reads which origin could not be attributed to either of them [9]. They also described an alternative classification strategy based on the Tophat splice junction mapper [15]. Firstly, human, mouse and xenograft RNA-Seq read sets were aligned to the host genome, then these were subsequently and independently aligned to the graft genome to finally post-process and classify the aligned reads into four types: host, graft, both and neither [9]. A method that simultaneously maps RNA-Seq sequencing reads to a merged reference combining both the host and graft transcriptomes has been described by Raskatov *et al* [10]. The authors argue that a minimum of 40% of the sequencing reads could be attributed to host-specific reads. The methodology described in our work could not identify such a high proportion of host-specific reads and agrees with what is been previously reported by the studies described in Conway *et al.* [9].

Our strategy expands the short-read aligner method [9] and uses widely used mapping tools, such as Bowtie-Tophat and BWA for classification purposes. By utilising a short-read aligner methodology, we successfully validated our strategy using the three main NGS techniques: RNA-Seq, exome-capture and low-coverage whole-genome sequence. In spite of being a more conservative approach than Xenome, *i. e*., the number of reads which fall into the *both* category is higher, our strategy is more robust when the certainty of the detections made is prioritized. Additionally, the customized modifications that can be made to the aligner parameters, such as seed length, number of mismatches in the seed and minimum mapping quality, could become an additional advantage supporting the robustness of the methodology. Although this approach was previously reported for RNA-Seq [9], [10], the authors did not describe a detailed workflow for the deep sequencing technologies mentioned above. Our strategy is based on short-read aligners with the advantage of flexible and customizable stringency. In addition, our classification/filtering process can be performed at the aligning stage, avoiding extra computing and storage requirements.

Rather than being a disadvantage, our data support the idea that the species-specific tumour-stromal interface innate to xenograft models allow us to more sensitively and specifically detect tumour-specific variants without the need for extra depth or complex algorithms needed to account for human stroma. The use of primary xenograft models derived from the overwhelming numbers of patients with inoperable solid tumours may therefore represent a useful platform for complex and informative NGS research.

## Supporting Information

Figure S1 **Copy number variation analysis. Complete human chromosome profile of the of CLH209 (A) cell line and a xenograft tumour derived from it (B).**
(PDF)

Figure S2 **Analysis of xenograft-specific variants. Local pairwise alignment of the human (hg19) and mouse (mm9) genomes compared against the sequencing read alignments of genomic regions surrounding the variants detected.** Representative detected variants are shown for *AQP7* (A), *APEH* (B), *MUC17* (C) and *MUC20* (D) genes. Genomic locations for the variants shown are described in supplementary Table 4 A. In the local pairwise alignments, vertical lines, and the number above them, represent sequence gaps and its length respectively; dots represent conserved human-mouse sequences. Variant position is highlighted by black parallel bars. Nucleotide residues are shown in red (thymine), blue (cytosine), green (adenine) and yellow (guanine). Heterozygous variants are indicated in the depth of coverage track and show both reference and alternative alleles. Forward and reverse sequencing reads are shown in pink and blue respectively. Sequence base mismatches are highlighted with its corresponding nucleotide colour.
(PDF)

Figure S3 **Analysis of xenograft-specific variants. Sequence depth of coverage and allele frequency comparisons between the xenograft and cell line samples.** Both samples were aligned to the human reference genome hg19. Representative detected variants are shown for *AQP7* (A), *APEH* (B), *MUC17* (C) and *MUC20* (D) genes. Genomic locations for the variants shown are described in supplementary Table 4 A. Variants position is highlighted by black parallel bars. Nucleotide residues are shown in red (thymine), blue (cytosine), green (adenine) and yellow (guanine). Heterozygous variants are indicated in the depth of coverage track and show both reference and alternative alleles. Forward and reverse sequencing reads are shown in pink and blue respectively. Sequence base mismatches are highlighted with its corresponding nucleotide colour.
(PDF)

Figure S4 **Analysis of cell line-specific variants. Sequence depth of coverage and allele frequency comparisons between the cell line and xenograft samples.** Both samples were aligned to the human reference genome hg19. Representative detected variants are shown for *HIF1A* (A), *TRIM22* (B), *GPR64* (C) and *MAML2* (D) genes. Genomic locations for the variants shown are described in supplementary Table 4 A. Variants position is highlighted by black parallel bars. Nucleotide residues are shown in red (thymine), blue (cytosine), green (adenine) and yellow (guanine). Heterozygous variants are indicated in the depth of coverage track and show both reference and alternative alleles. Forward and reverse sequencing reads are shown in pink and blue respectively. Sequence base mismatches are highlighted with its corresponding nucleotide color.
(PDF)

Table S1 **Summary of the total and post QA/QC number of sequenced reads from each NGS experiment performed.** Control: peripheral blood BL209, Cell line: NCI-H209; Xenograft: xenograft sample derived from the NCI-H209 cell line. LX22, LX33 and LX33: SCLC primary xenograft lines LX22, LX33 and LX36. The number of mapped reads for the xenograft samples are human-specific only. PE: pair-ends.
(PDF)

Table S2 **Summary of the alignments statistics of each NGS experiment performed.** LX22, LX33 and LX33: SCLC primary xenograft lines LX22, LX33 and LX36.Control: peripheral blood BL209, Cell line: NCI-H209; Xenograft: xenograft sample derived from the NCI-H209 cell line. The number of mapped reads for the xenograft samples are human-specific only. For the exome capture and low-coverage whole genome analyses of the xenograft sample concordantly paired reads were analyzed.
(PDF)

Table S3 **Summary of the depth of coverage obtained for the exome capture (A) and low pass whole genome (B) experiments.** Control: peripheral blood BL209, Cell line: NCI-H209; Xenograft: xenograft sample derived from the NCI-H209 cell line. The results of the xenograft sample only represent those reads that were human specific. Depth of coverage was estimated on whole reads.
(PDF)

Table S4 **Table describing sample-specific single nucleotide variants.** (A) Xenograft-specific non-synonymous coding variants. (B) Cell line specific non-synonymous coding variants. For sample-specific variants, approximate read depth is shown (reads with MQ = 255 or with bad mates were filtered). Read and allelic depth of the sample were the variant was not identified were calculated on de-duplicated reads. NSC: non-synonymous coding variant.
(PDF)

Dataset S1 **Detected copy number variations in the NCI-H209 cell line sample.**
(PDF)

Dataset S2 **Detected copy number variations in the xenograft sample derived from the NCI-H209 cell line.**
(PDF)

Dataset S3 I**nter- and intrachromosomal rearrangements common to both the NCI-H209 cell line and its derived xenograft sample.**
(PDF)

Dataset S4 **Somatic variants detected in both the cell line and xenograft samples.**
(PDF)

Dataset S5 **SNPs effects common to both the cell line and xenograft samples (only one transcript of the moderate and high impact effects are reported.**
(PDF)
