# Supplementary material for: Next-Generation Sequence Analysis of Cancer Xenograft Models
Source: PLoS One. 2013 Sep 26;8(9):e74432. doi: 10.1371/journal.pone.0074432 (PMC3784448; doi:10.1371/journal.pone.0074432)
Supplement: Figure S1 — Copy number variation analysis. Complete human chromosome profile of the of CLH209 (A) cell line and a xenograft tumour derived from it (B). (PDF) [file pone.0074432.s001.pdf]

A

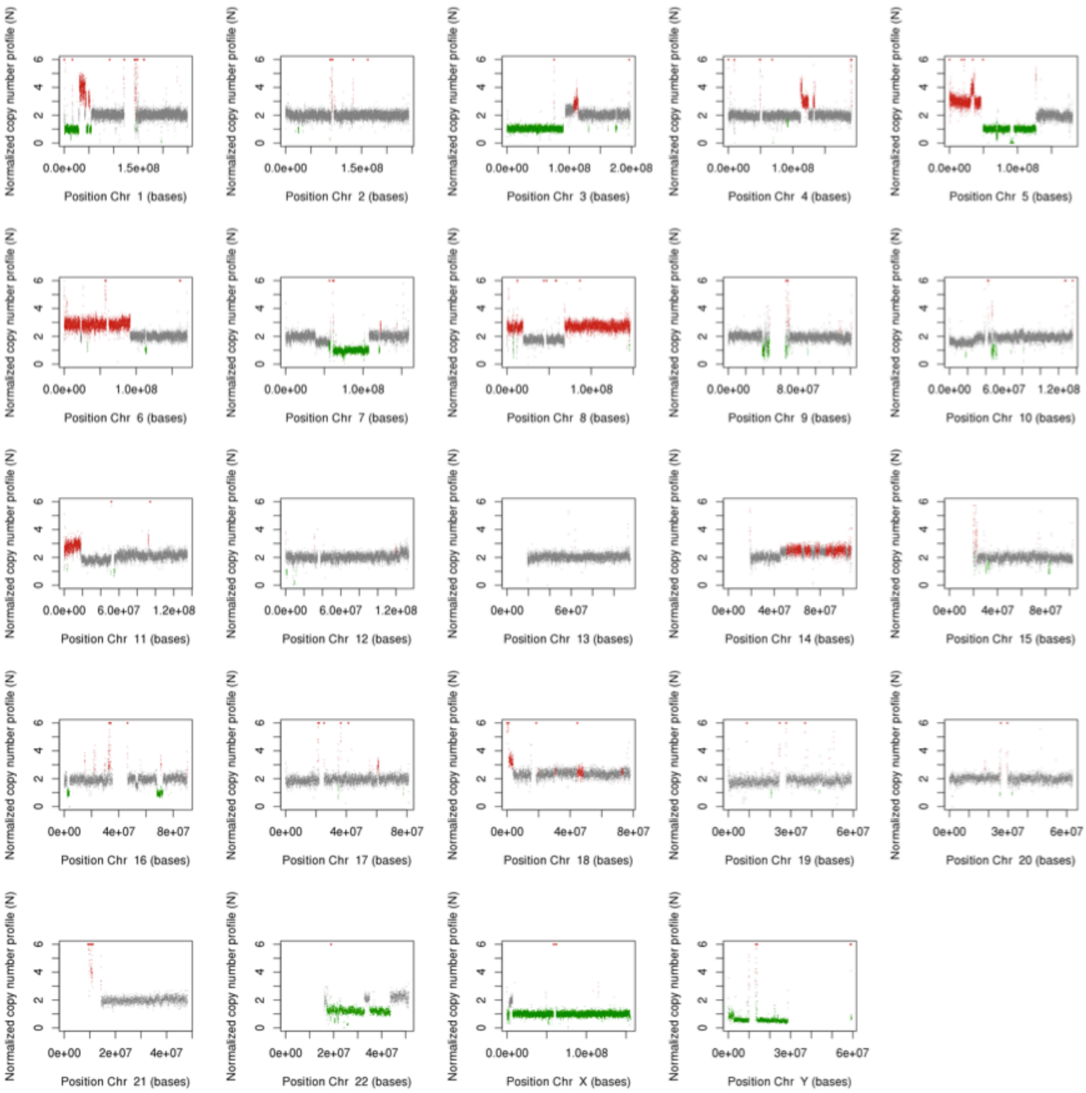

Normalized copy number profile (N)
Position Chr 1 (bases)
Position Chr 2 (bases)
Position Chr 3 (bases)
Position Chr 4 (bases)
Position Chr 5 (bases)
Position Chr 6 (bases)
Position Chr 7 (bases)
Position Chr 8 (bases)
Position Chr 9 (bases)
Position Chr 10 (bases)
Position Chr 11 (bases)
Position Chr 12 (bases)
Position Chr 13 (bases)
Position Chr 14 (bases)
Position Chr 15 (bases)
Position Chr 16 (bases)
Position Chr 17 (bases)
Position Chr 18 (bases)
Position Chr 19 (bases)
Position Chr 20 (bases)
Position Chr 21 (bases)
Position Chr 22 (bases)
Position Chr X (bases)
Position Chr Y (bases)

B

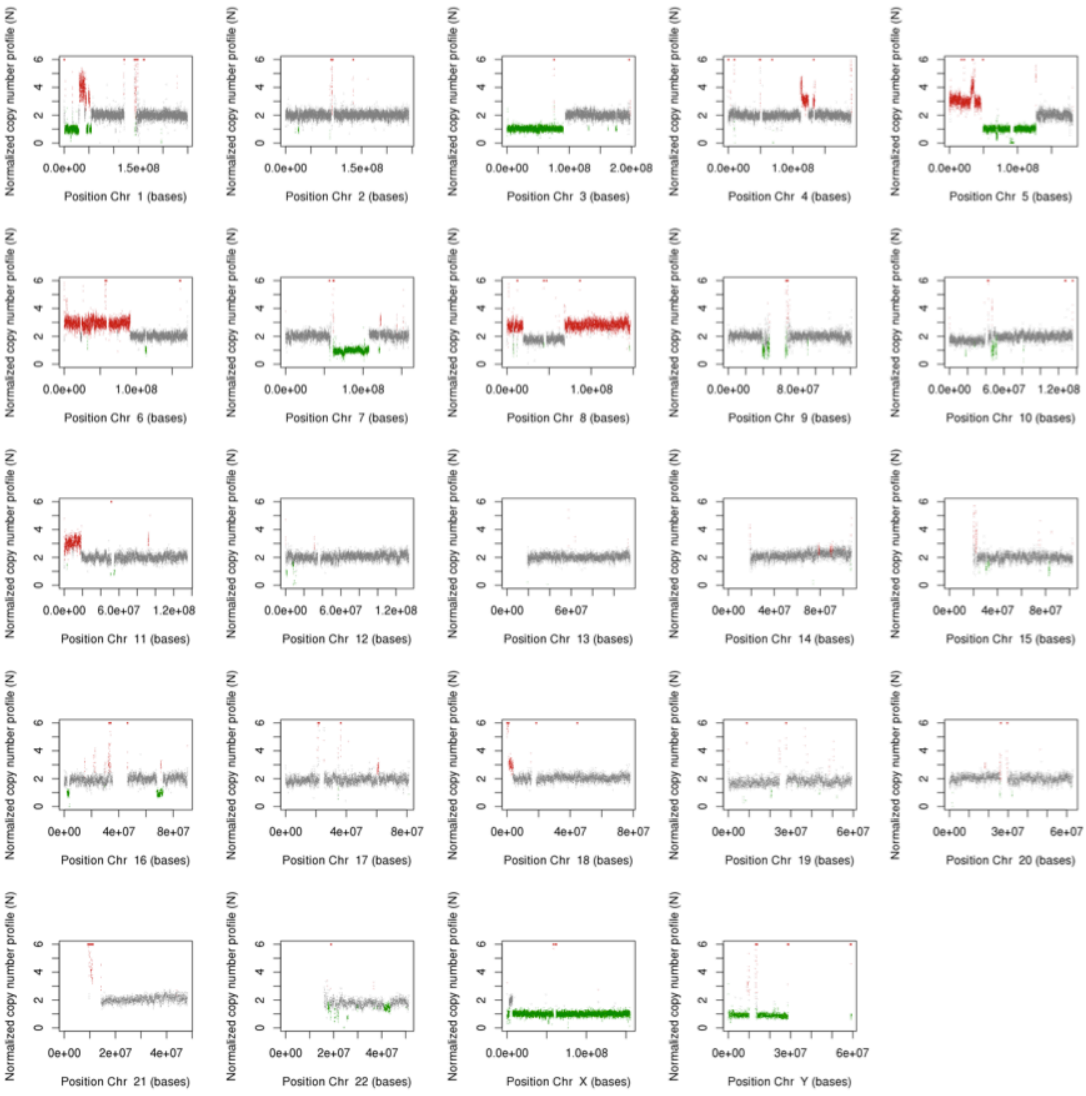

Figure S1. Copy number variation analysis. Complete human chromosome profile of the of CLH209 (A) cell line and a xenograft tumour derived from it (B).
