# Supplementary material for: Next-Generation Sequence Analysis of Cancer Xenograft Models
Source: PLoS One. 2013 Sep 26;8(9):e74432. doi: 10.1371/journal.pone.0074432 (PMC3784448; doi:10.1371/journal.pone.0074432)
Supplement: Figure S2 — Analysis of xenograft-specific variants. Local pairwise alignment of the human (hg19) and mouse (mm9) genomes compared against the sequencing read alignments of genomic regions surrounding the variants detected. Representative detected variants are shown for AQP7 (A), APEH (B), MUC17 (C) and MUC20 (D) genes. Genomic locations for the variants shown are described in supplementary Table 4 A. In the local pairwise alignments, vertical lines, and the number above them, represent sequence gaps and its length respectively; dots represent conserved human-mouse sequences. Variant position is highlighted by black parallel bars. Nucleotide residues are shown in red (thymine), blue (cytosine), green (adenine) and yellow (guanine). Heterozygous variants are indicated in the depth of coverage track and show both reference and alternative alleles. Forward and reverse sequencing reads are shown in pink and blue respectively. Sequence base mismatches are highlighted with its corresponding nucleotide colour. (PDF) [file pone.0074432.s002.pdf]

A

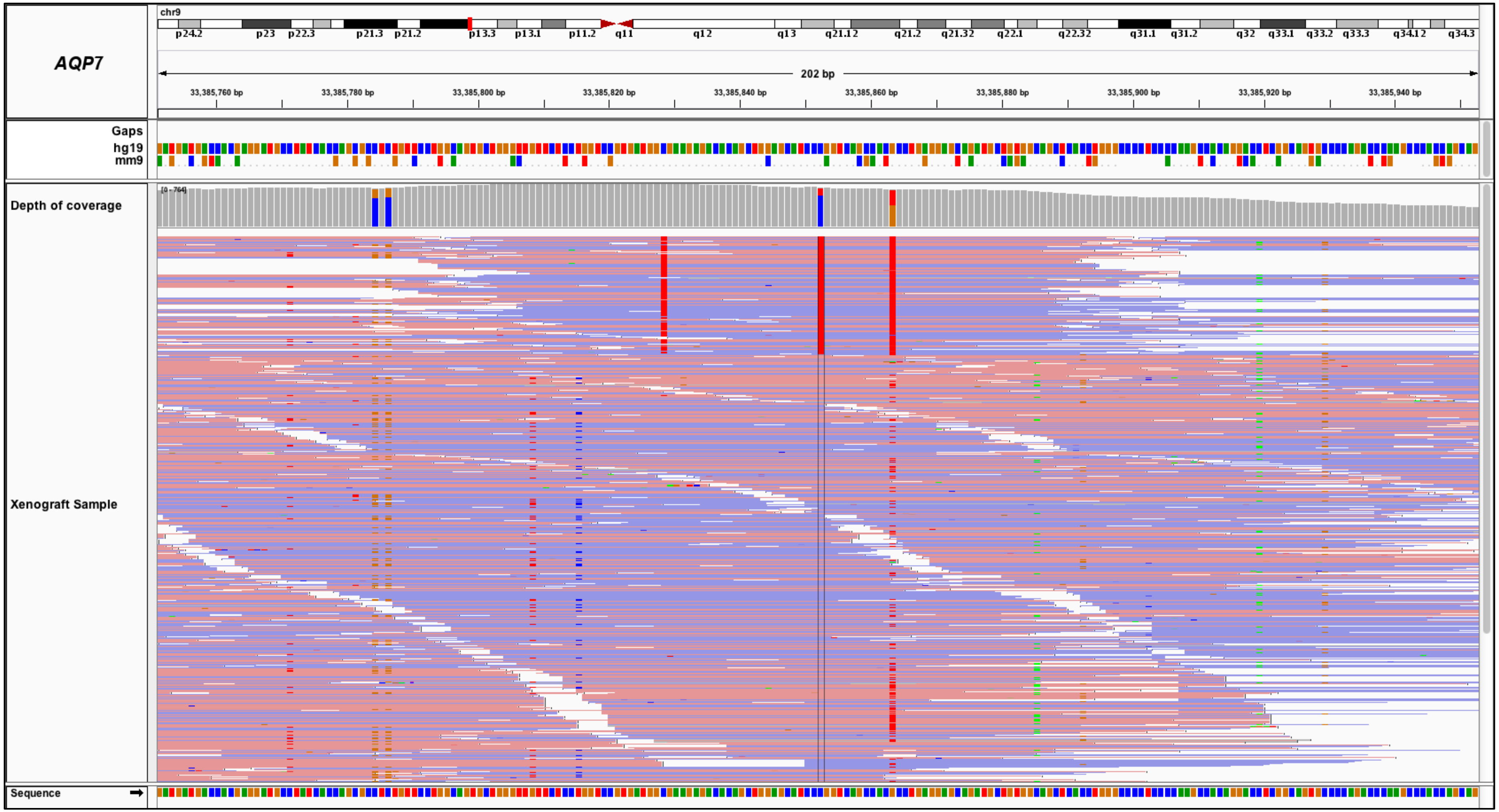

AQP7
chr9
p24.2
p23
p22.3
p21.3
p21.2
p13.3
p13.1
p11.2
q11
q12
q13
q21.12
q21.2
q21.32
q22.1
q22.32
q31.1
q31.2
q32
q33.1
q33.2
q33.3
q34.12
q34.3
202 bp
33,385,760 bp
33,385,780 bp
33,385,800 bp
33,385,820 bp
33,385,840 bp
33,385,860 bp
33,385,880 bp
33,385,900 bp
33,385,920 bp
33,385,940 bp
Gaps
hg19
mm9
Depth of coverage
Xenograft Sample
Sequence

B

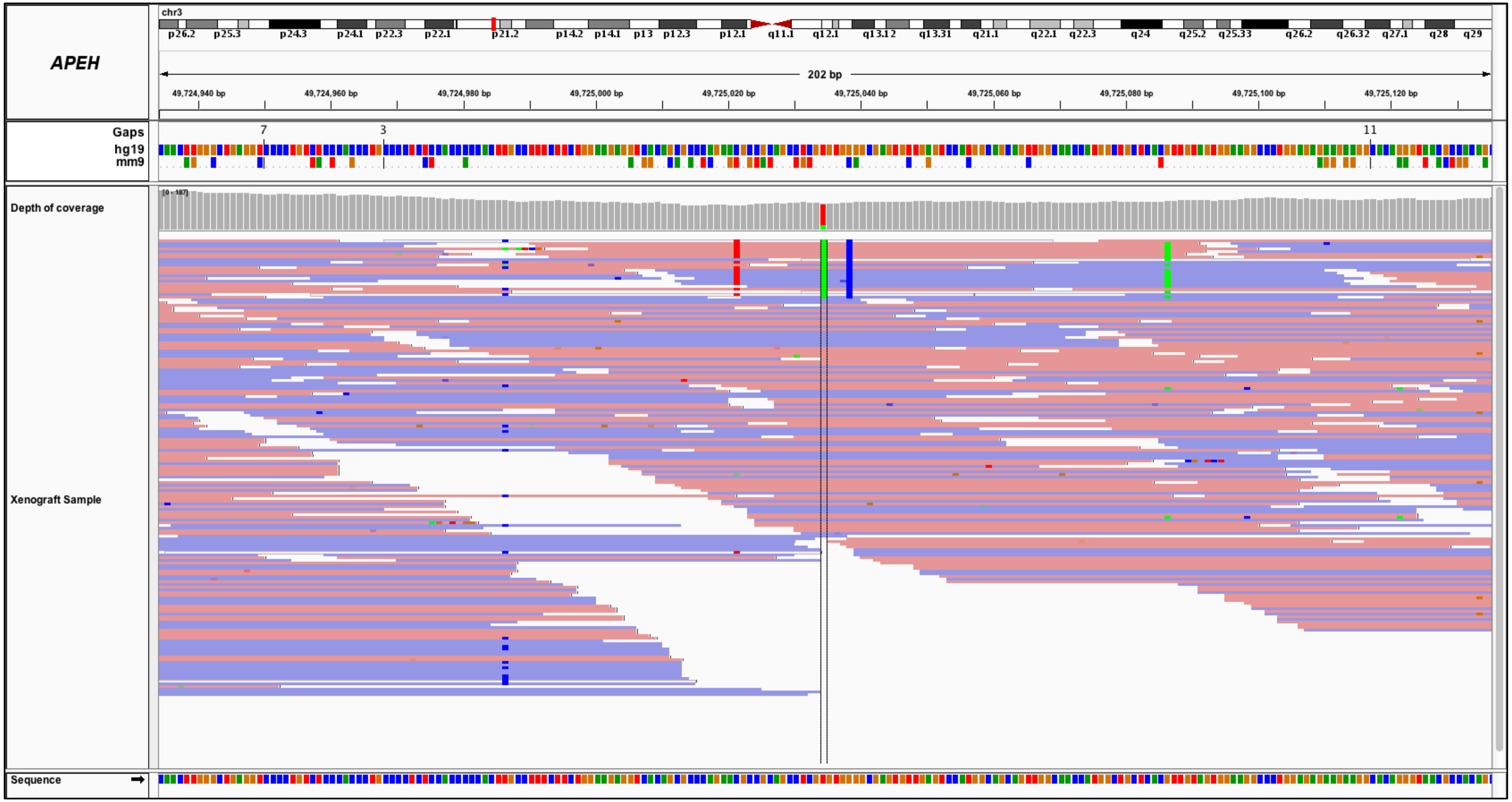

APEH
chr3
p26.2
p25.3
p24.3
p24.1
p22.3
p22.1
p21.2
p14.2
p14.1
p13
p12.3
p12.1
q11.1
q12.1
q13.12
q13.31
q21.1
q22.1
q22.3
q24
q25.2
q25.33
q26.2
q26.32
q27.1
q28
q29
202 bp
49,724,940 bp
49,724,960 bp
49,724,980 bp
49,725,000 bp
49,725,020 bp
49,725,040 bp
49,725,060 bp
49,725,080 bp
49,725,100 bp
49,725,120 bp
Gaps
hg19
mm9
7
3
11
Depth of coverage
Xenograft Sample
Sequence

C

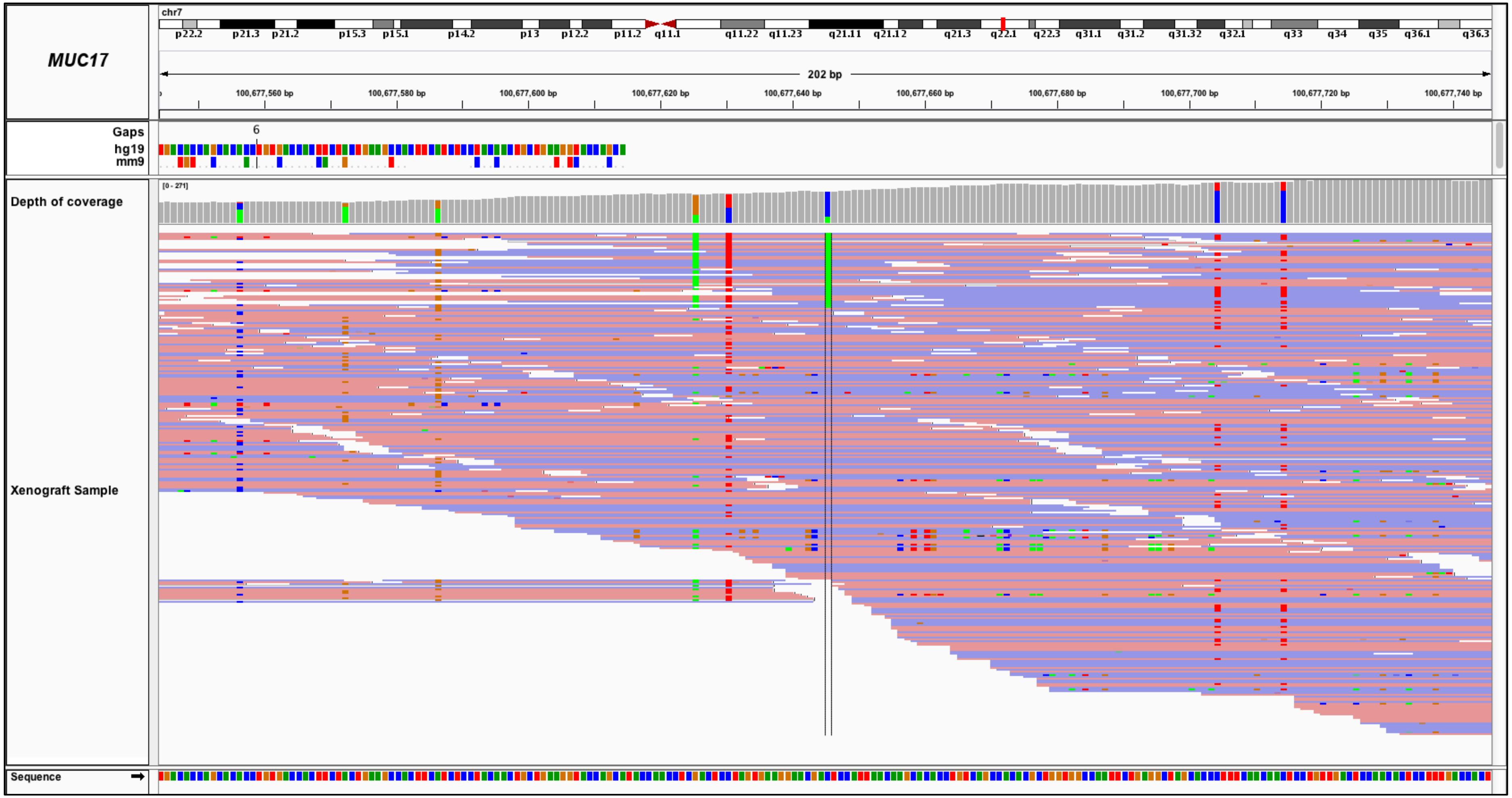

MUC17
chr7
p22.2 p21.3 p21.2 p15.3 p15.1 p14.2 p13 p12.2 p11.2 q11.1 q11.22 q11.23 q21.11 q21.12 q21.3 q22.1 q22.3 q31.1 q31.2 q31.32 q32.1 q33 q34 q35 q36.1 q36.3
202 bp
100,677,560 bp
100,677,580 bp
100,677,600 bp
100,677,620 bp
100,677,640 bp
100,677,660 bp
100,677,680 bp
100,677,700 bp
100,677,720 bp
100,677,740 bp
Gaps
6
hg19
mm9
Depth of coverage
[0 - 271]
Xenograft Sample
Sequence

D

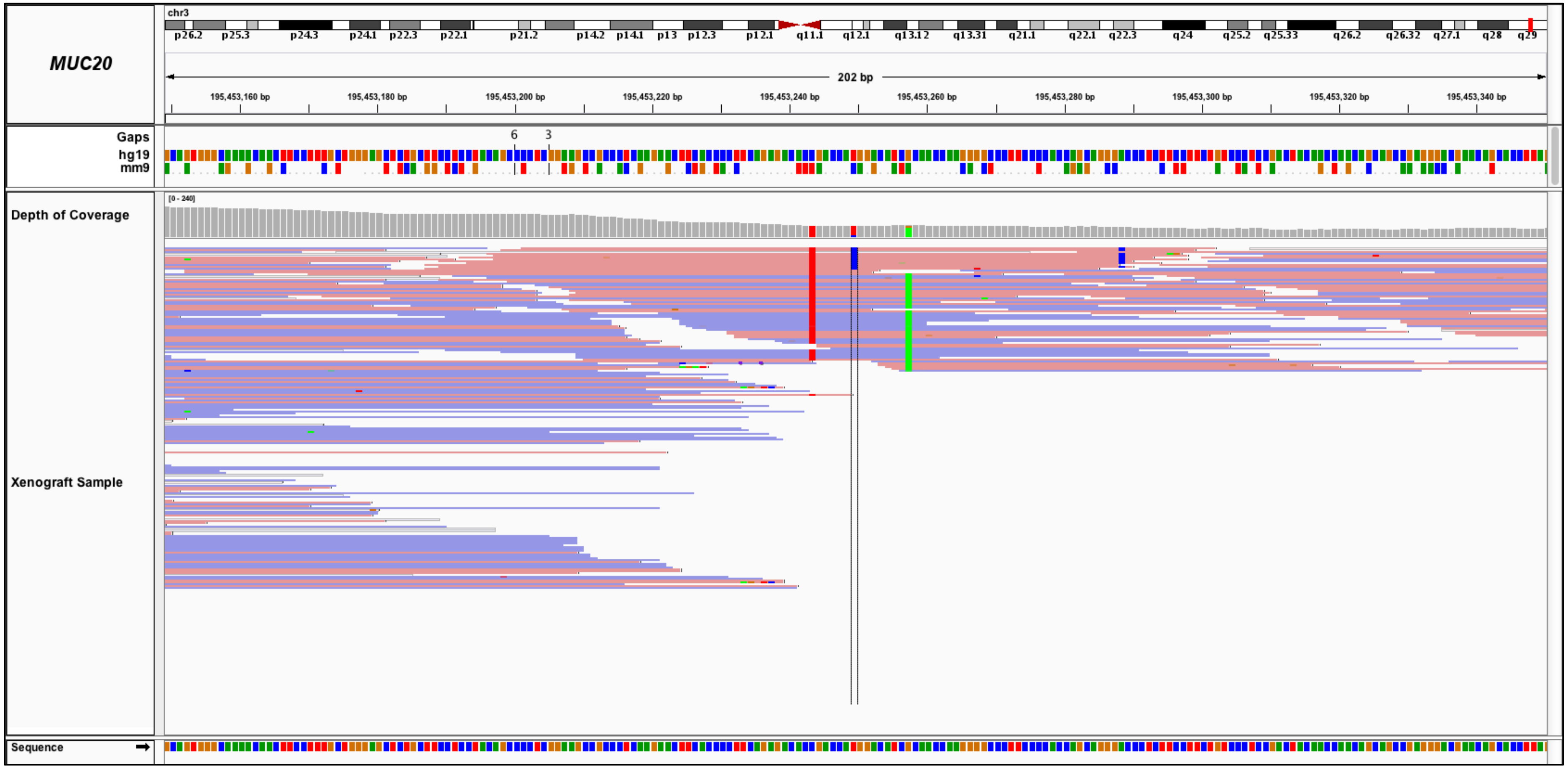

Figure S2. Analysis of xenograft-specific variants. Local pairwise alignment of the human (hg19) and mouse (mm9) genomes compared against the sequencing read alignments of genomic regions surrounding the variants detected. Representative detected variants are shown for *AQP7* (A), *APEH* (B), *MUC17* (C) and *MUC20* (D) genes. Genomic locations for the variants shown are described in supplementary Table 4 A. In the local pairwise alignments, vertical lines, and the number above them, represent sequence gaps and its length respectively; dots represent conserved human-mouse sequences. Variant position is highlighted by black parallel bars. Nucleotide residues are shown in red (thymine), blue (cytosine), green (adenine) and yellow (guanine). Heterozygous variants are indicated in the depth of coverage track and show both reference and alternative alleles. Forward and reverse sequencing reads are shown in pink and blue respectively. Sequence base mismatches are highlighted with its corresponding nucleotide color.
