# Supplementary material for: Next-Generation Sequence Analysis of Cancer Xenograft Models
Source: PLoS One. 2013 Sep 26;8(9):e74432. doi: 10.1371/journal.pone.0074432 (PMC3784448; doi:10.1371/journal.pone.0074432)
Supplement: Figure S3 — Analysis of xenograft-specific variants. Sequence depth of coverage and allele frequency comparisons between the xenograft and cell line samples. Both samples were aligned to the human reference genome hg19. Representative detected variants are shown for AQP7 (A), APEH (B), MUC17 (C) and MUC20 (D) genes. Genomic locations for the variants shown are described in supplementary Table 4 A. Variants position is highlighted by black parallel bars. Nucleotide residues are shown in red (thymine), blue (cytosine), green (adenine) and yellow (guanine). Heterozygous variants are indicated in the depth of coverage track and show both reference and alternative alleles. Forward and reverse sequencing reads are shown in pink and blue respectively. Sequence base mismatches are highlighted with its corresponding nucleotide colour. (PDF) [file pone.0074432.s003.pdf]

A

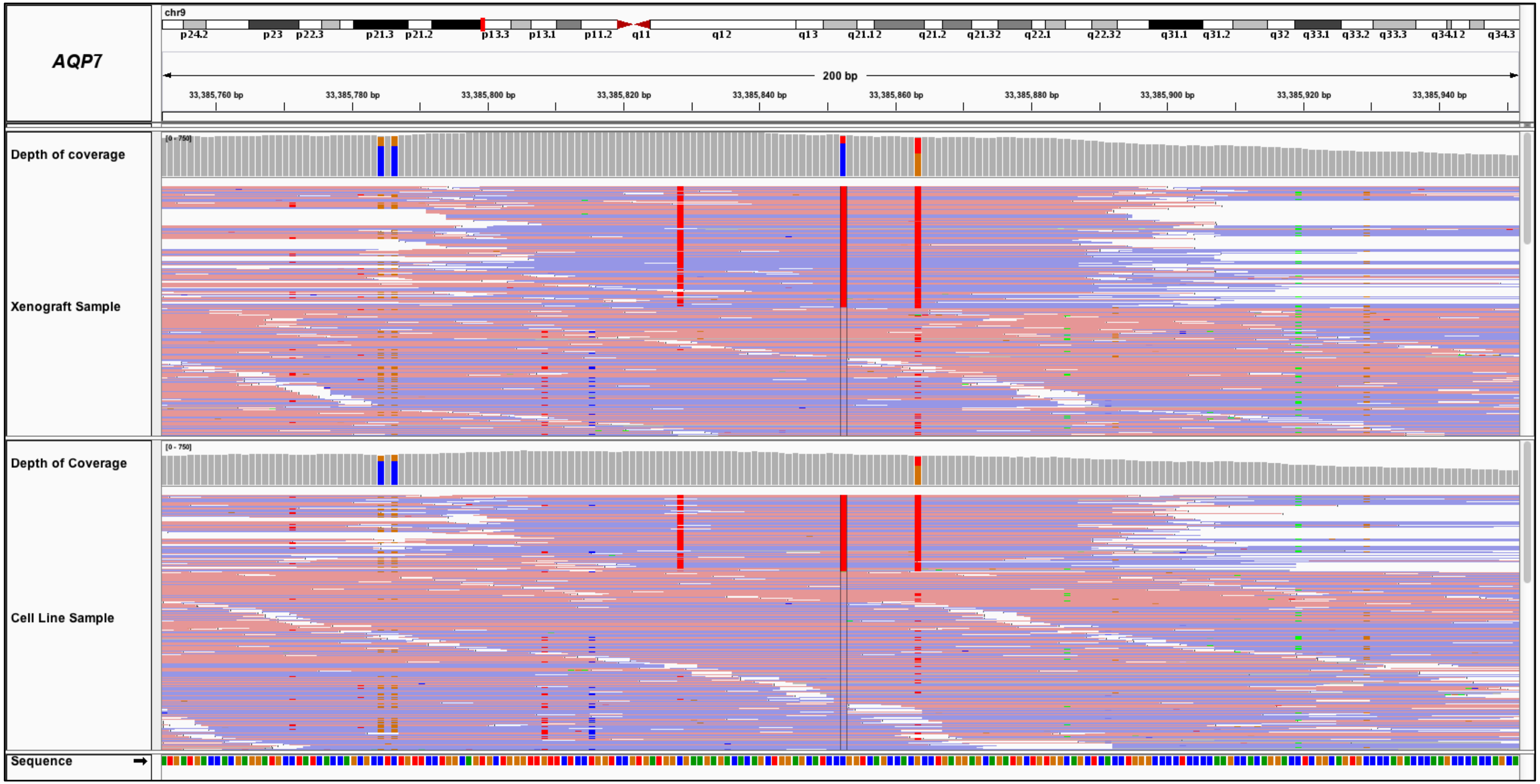
AQP7
chr9
p24.2
p23
p22.3
p21.3
p21.2
p13.3
p13.1
p11.2
q11
q12
q13
q21.12
q21.2
q21.32
q22.1
q22.32
q31.1
q31.2
q32
q33.1
q33.2
q33.3
q34.12
q34.3
200 bp
33,385,760 bp
33,385,780 bp
33,385,800 bp
33,385,820 bp
33,385,840 bp
33,385,860 bp
33,385,880 bp
33,385,900 bp
33,385,920 bp
33,385,940 bp
Depth of coverage
[0 - 750]
Xenograft Sample
Depth of Coverage
[0 - 750]
Cell Line Sample
Sequence

B

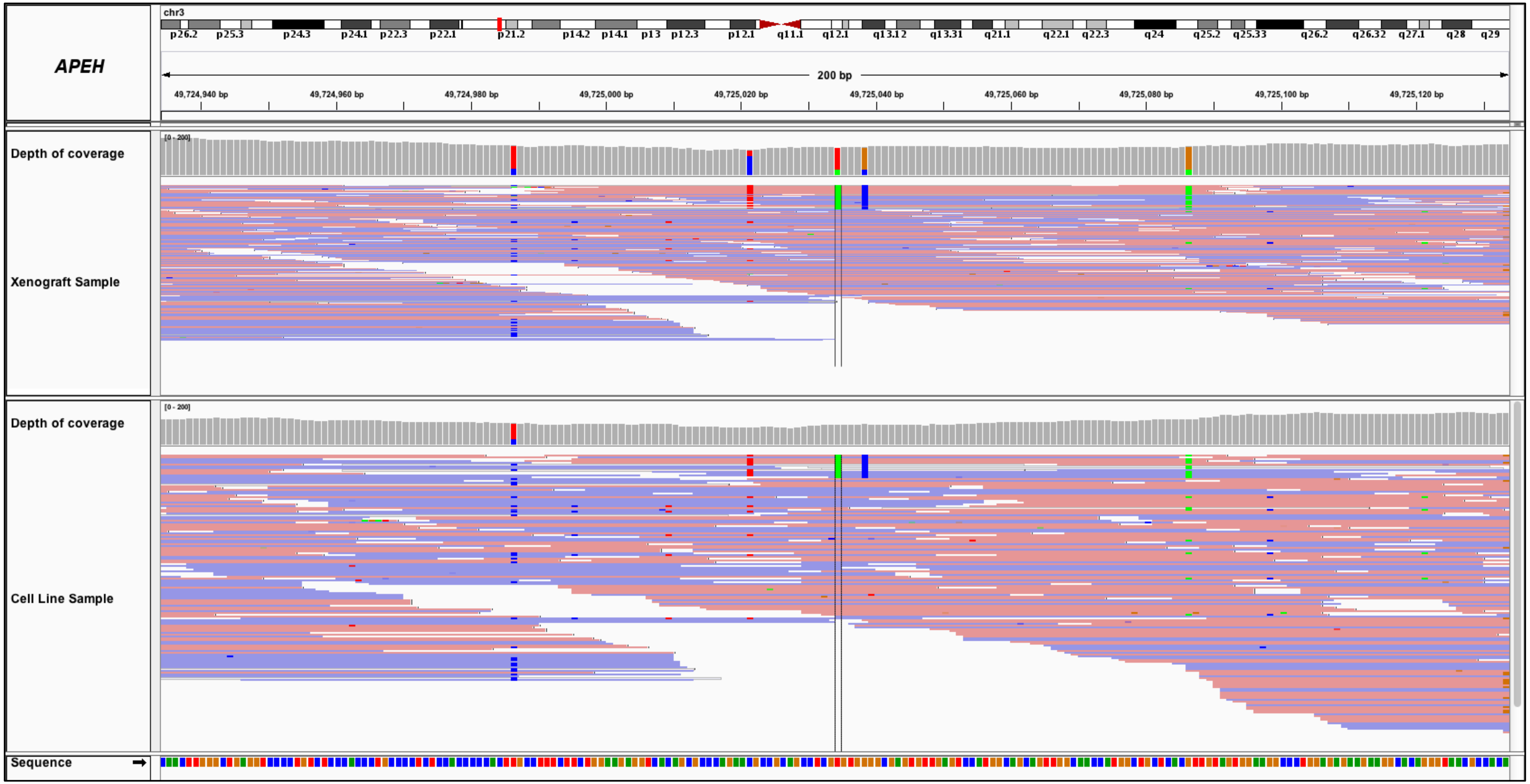
APEH
chr3
p26.2 p25.3 p24.3 p24.1 p22.3 p22.1 p21.2 p14.2 p14.1 p13 p12.3 p12.1 q11.1 q12.1 q13.12 q13.31 q21.1 q22.1 q22.3 q24 q25.2 q25.33 q26.2 q26.32 q27.1 q28 q29
200 bp
49,724,940 bp
49,724,960 bp
49,724,980 bp
49,725,000 bp
49,725,020 bp
49,725,040 bp
49,725,060 bp
49,725,080 bp
49,725,100 bp
49,725,120 bp
Depth of coverage
[0 - 200]
Xenograft Sample
Depth of coverage
[0 - 200]
Cell Line Sample
Sequence

C

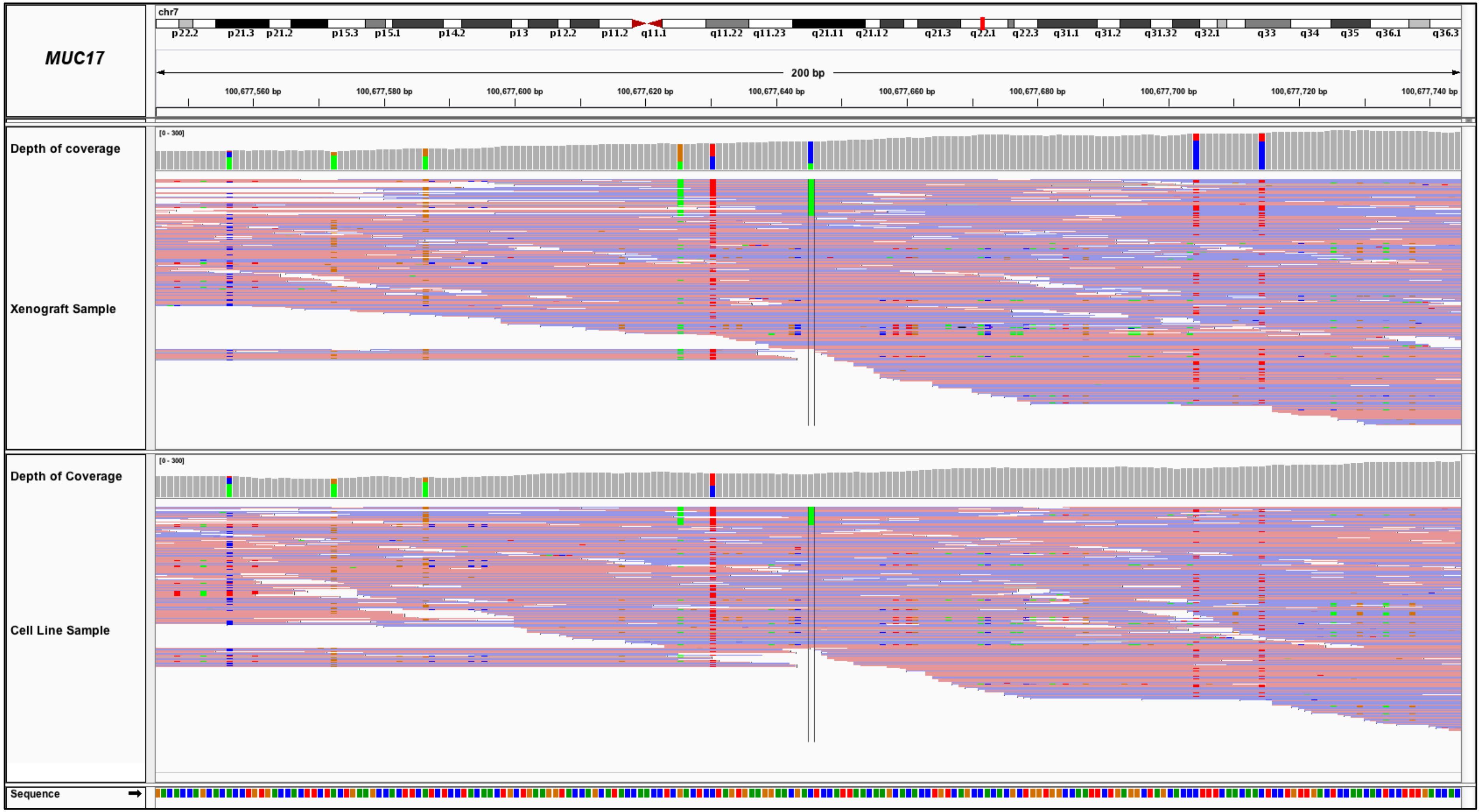

MUC17
chr7
p22.2 p21.3 p21.2 p15.3 p15.1 p14.2 p13 p12.2 p11.2 q11.1 q11.22 q11.23 q21.11 q21.12 q21.3 q22.1 q22.3 q31.1 q31.2 q31.32 q32.1 q33 q34 q35 q36.1 q36.3
200 bp
100,677,560 bp
100,677,580 bp
100,677,600 bp
100,677,620 bp
100,677,640 bp
100,677,660 bp
100,677,680 bp
100,677,700 bp
100,677,720 bp
100,677,740 bp
Depth of coverage
[0 - 300]
Xenograft Sample
Depth of Coverage
[0 - 300]
Cell Line Sample
Sequence

D

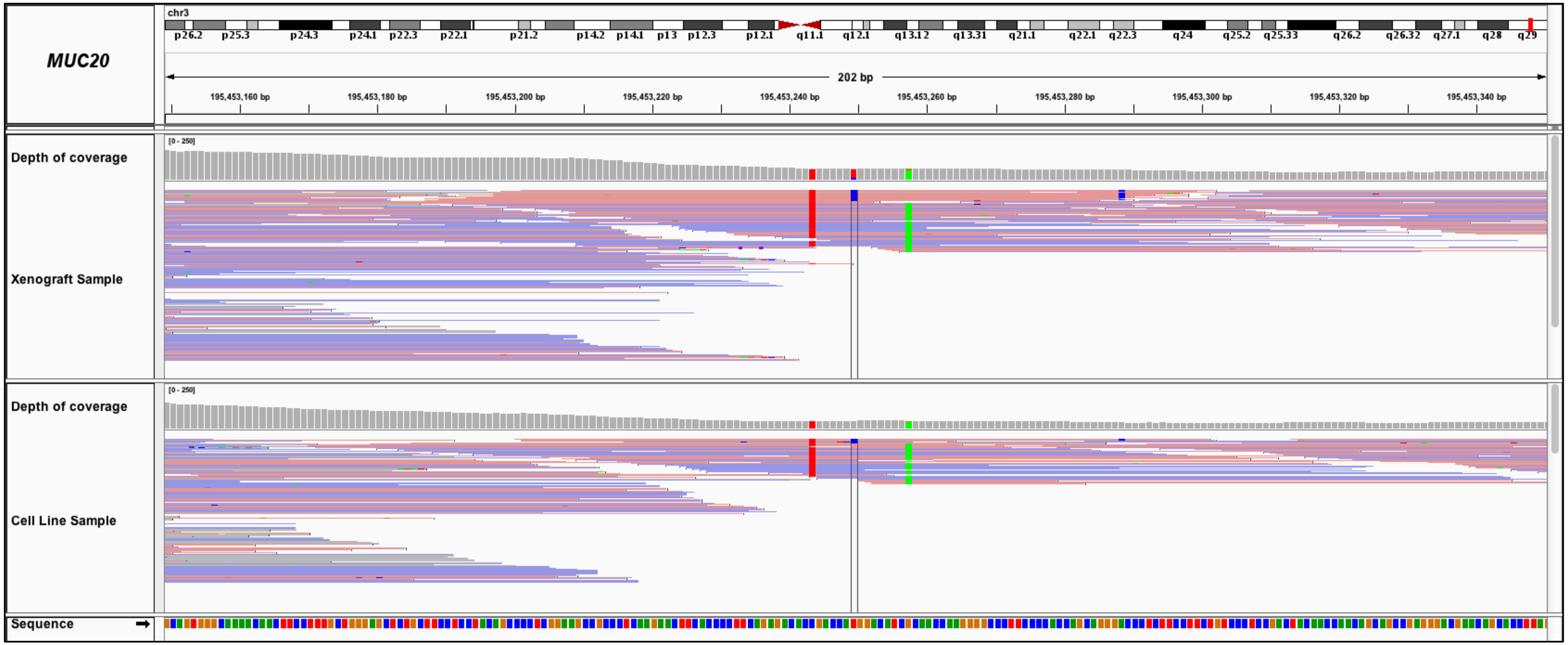

Figure S3. Analysis of xenograft-specific variants. Sequence depth of coverage and allele frequency comparisons between the xenograft and cell line samples. Both samples were aligned to the human reference genome hg19. Representative detected variants are shown for *AQP7* (A), *APEH* (B), *MUC17* (C) and *MUC20* (D) genes. Genomic locations for the variants shown are described in supplementary Table 4 A. Variants position is highlighted by black parallel bars. Nucleotide residues are shown in red (thymine), blue (cytosine), green (adenine) and yellow (guanine). Heterozygous variants are indicated in the depth of coverage track and show both reference and alternative alleles. Forward and reverse sequencing reads are shown in pink and blue respectively. Sequence base mismatches are highlighted with its corresponding nucleotide color.
