# Supplementary material for: Next-Generation Sequence Analysis of Cancer Xenograft Models
Source: PLoS One. 2013 Sep 26;8(9):e74432. doi: 10.1371/journal.pone.0074432 (PMC3784448; doi:10.1371/journal.pone.0074432)
Supplement: Figure S4 — Analysis of cell line-specific variants. Sequence depth of coverage and allele frequency comparisons between the cell line and xenograft samples. Both samples were aligned to the human reference genome hg19. Representative detected variants are shown for HIF1A (A), TRIM22 (B), GPR64 (C) and MAML2 (D) genes. Genomic locations for the variants shown are described in supplementary Table 4 A. Variants position is highlighted by black parallel bars. Nucleotide residues are shown in red (thymine), blue (cytosine), green (adenine) and yellow (guanine). Heterozygous variants are indicated in the depth of coverage track and show both reference and alternative alleles. Forward and reverse sequencing reads are shown in pink and blue respectively. Sequence base mismatches are highlighted with its corresponding nucleotide color. (PDF) [file pone.0074432.s004.pdf]

A

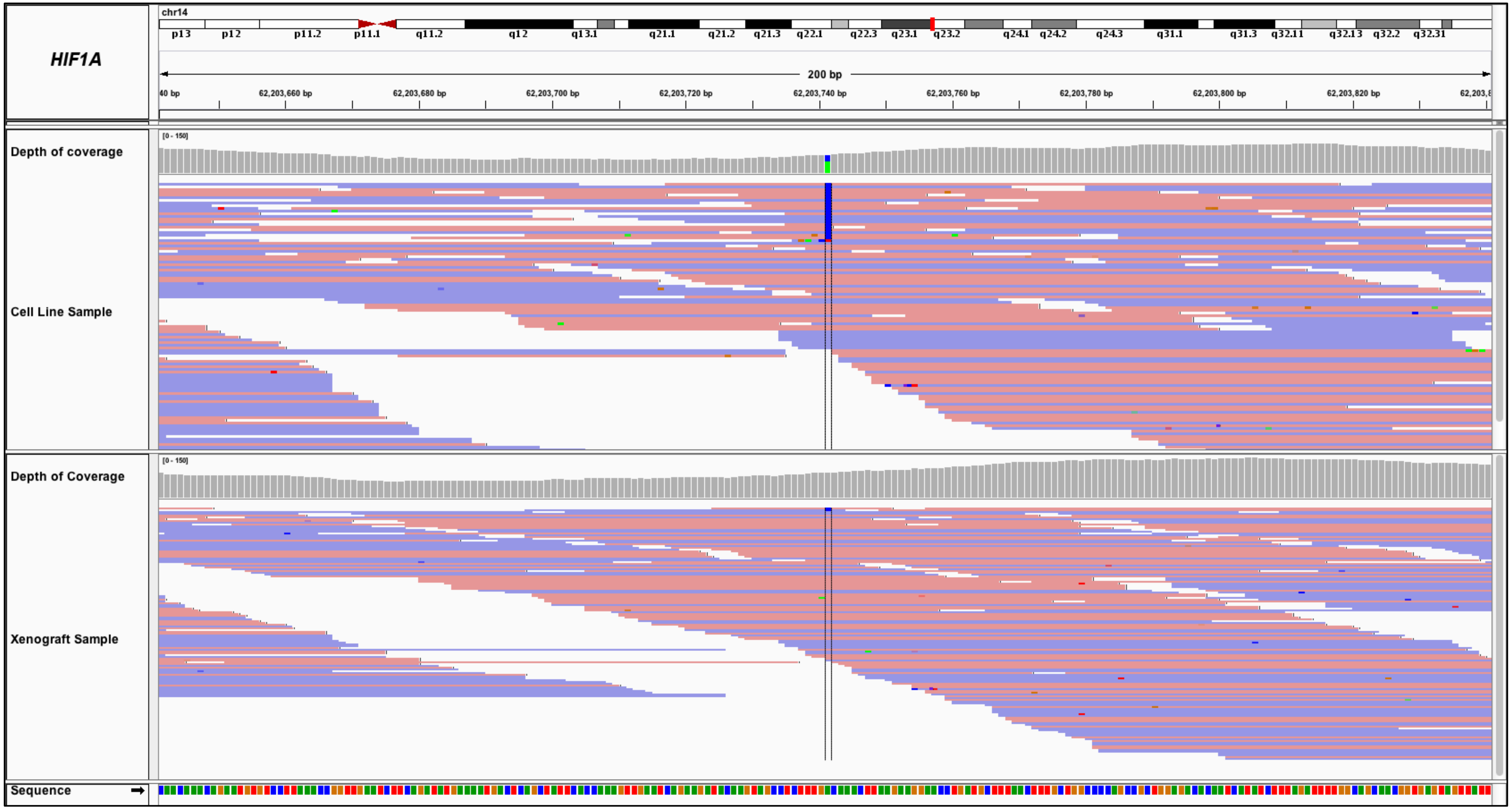
HIF1A
chr14
p13
p12
p11.2
p11.1
q11.2
q12
q13.1
q21.1
q21.2
q21.3
q22.1
q22.3
q23.1
q23.2
q24.1
q24.2
q24.3
q31.1
q31.3
q32.11
q32.13
q32.2
q32.31
200 bp
62,203,660 bp
62,203,680 bp
62,203,700 bp
62,203,720 bp
62,203,740 bp
62,203,760 bp
62,203,780 bp
62,203,800 bp
62,203,820 bp
Depth of coverage
[0 - 150]
Cell Line Sample
Depth of Coverage
[0 - 150]
Xenograft Sample
Sequence

B

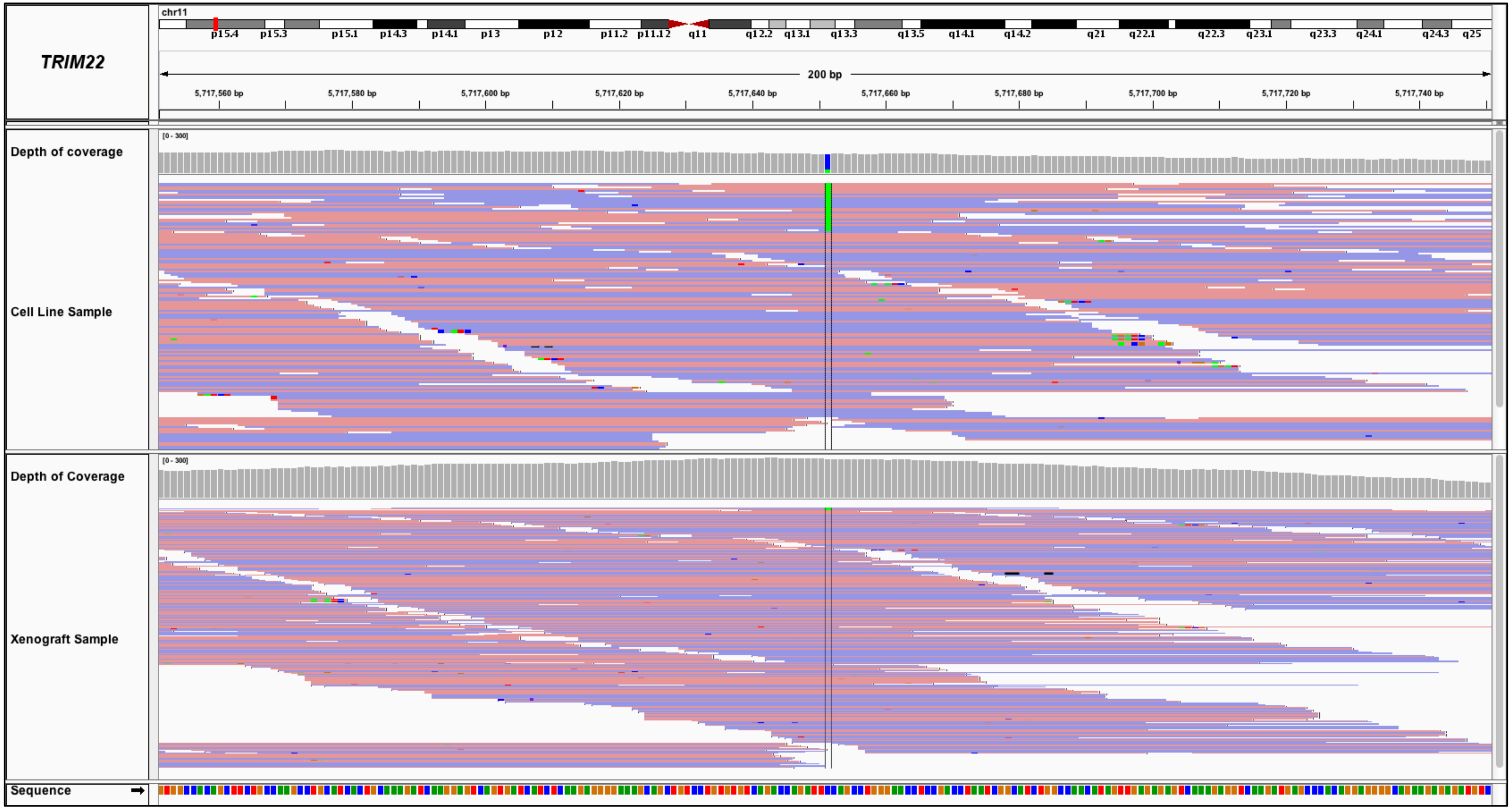
TRIM22
chr11
p15.4
p15.3
p15.1
p14.3
p14.1
p13
p12
p11.2
p11.12
q11
q12.2
q13.1
q13.3
q13.5
q14.1
q14.2
q21
q22.1
q22.3
q23.1
q23.3
q24.1
q24.3
q25
200 bp
5,717,560 bp
5,717,580 bp
5,717,600 bp
5,717,620 bp
5,717,640 bp
5,717,660 bp
5,717,680 bp
5,717,700 bp
5,717,720 bp
5,717,740 bp
Depth of coverage
[0 - 300]
Cell Line Sample
Depth of Coverage
[0 - 300]
Xenograft Sample
Sequence

C

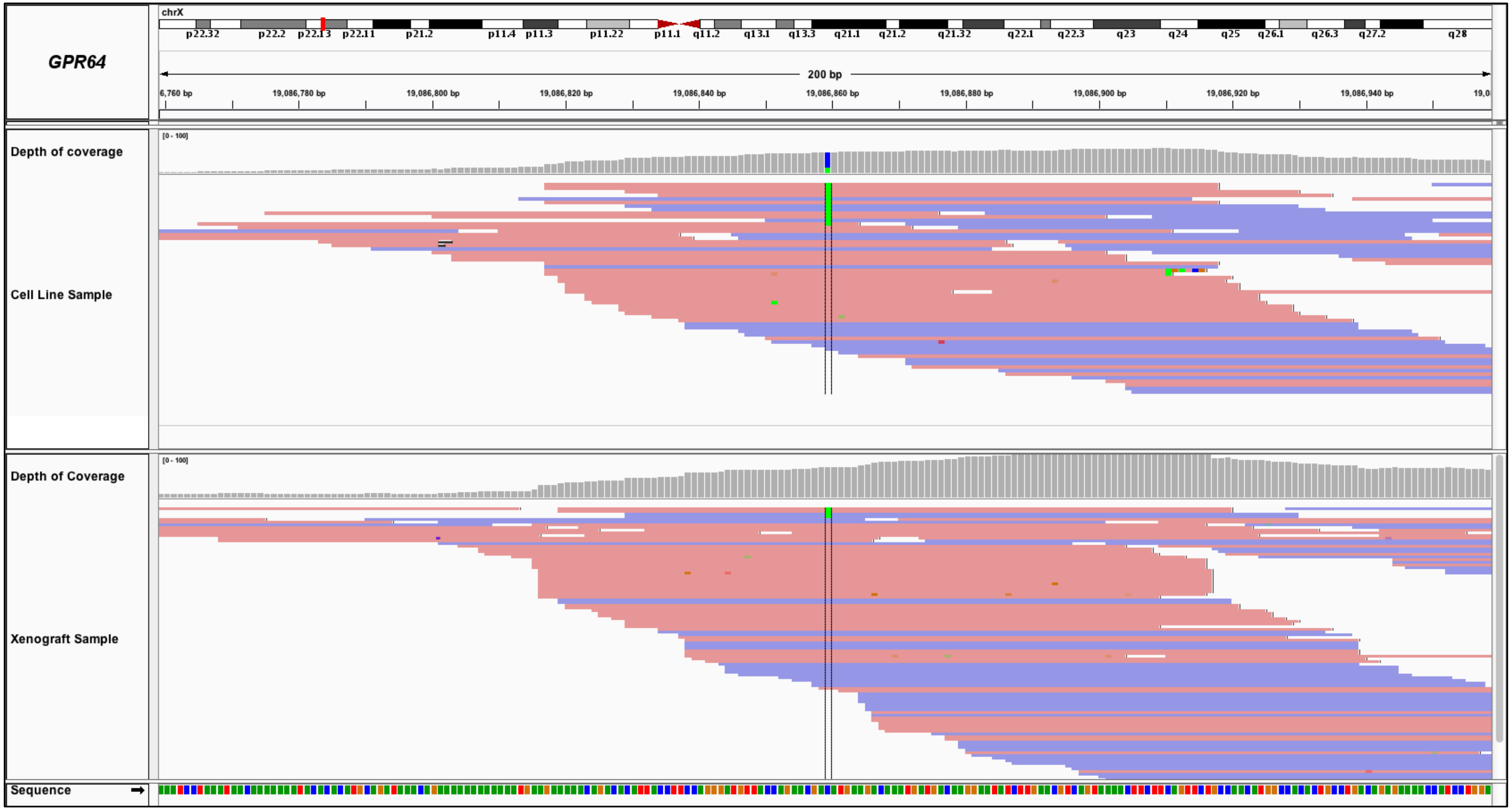

GPR64
chrX
p22.32
p22.2
p22.13
p22.11
p21.2
p11.4
p11.3
p11.22
p11.1
q11.2
q13.1
q13.3
q21.1
q21.2
q21.32
q22.1
q22.3
q23
q24
q25
q26.1
q26.3
q27.2
q28
200 bp
19,086,780 bp
19,086,800 bp
19,086,820 bp
19,086,840 bp
19,086,860 bp
19,086,880 bp
19,086,900 bp
19,086,920 bp
19,086,940 bp
Depth of coverage
[0 - 100]
Cell Line Sample
Depth of Coverage
[0 - 100]
Xenograft Sample
Sequence

D

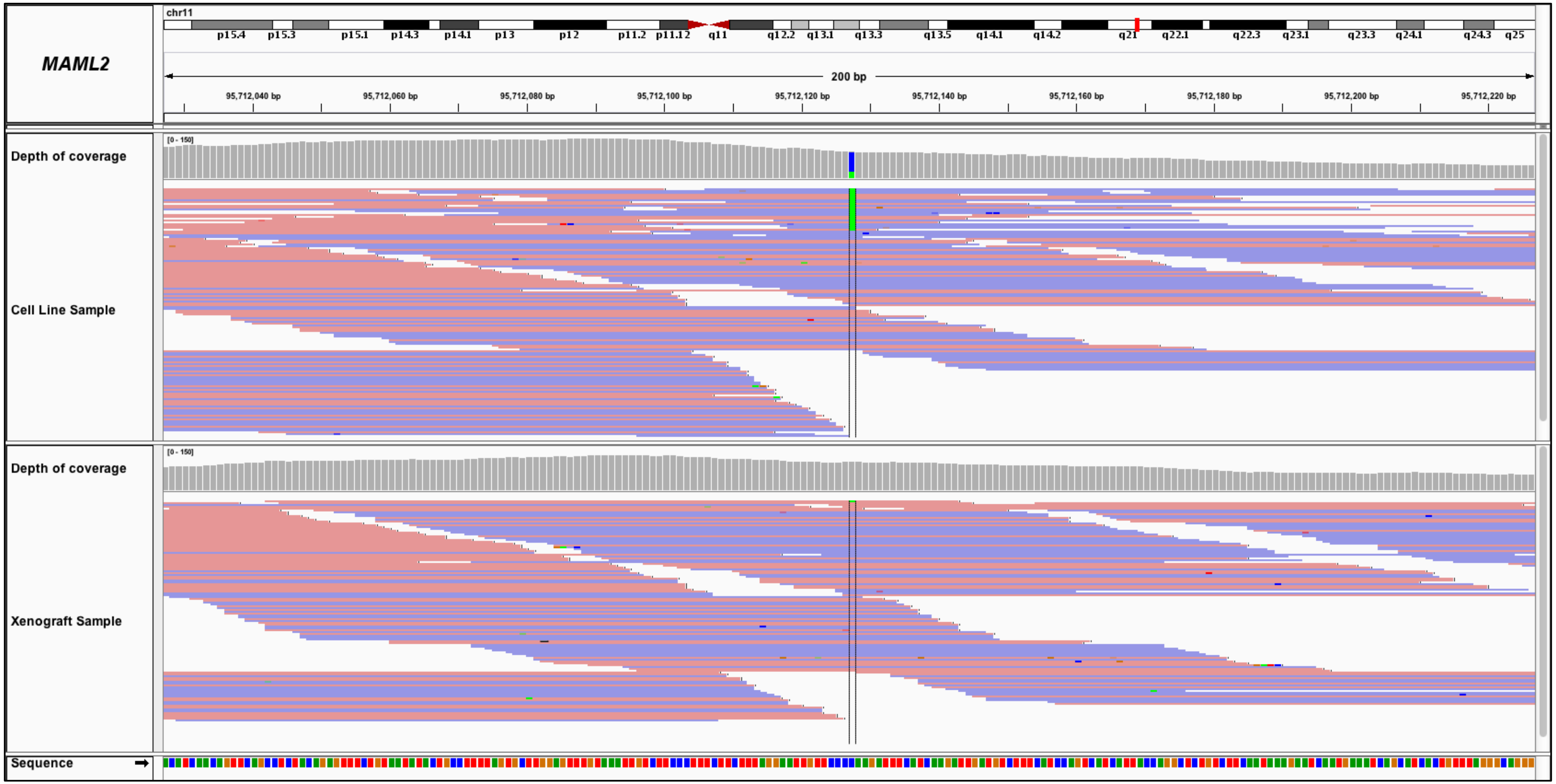

Figure S4. Analysis of cell line-specific variants. Sequence depth of coverage and allele frequency comparisons between the cell line and xenograft samples. Both samples were aligned to the human reference genome hg19. Representative detected variants are shown for *HIF1A* (A), *TRIM22* (B), *GPR64* (C) and *MAML2* (D) genes. Genomic locations for the variants shown are described in supplementary Table 4 A. Variants position is highlighted by black parallel bars. Nucleotide residues are shown in red (thymine), blue (cytosine), green (adenine) and yellow (guanine). Heterozygous variants are indicated in the depth of coverage track and show both reference and alternative alleles. Forward and reverse sequencing reads are shown in pink and blue respectively. Sequence base mismatches are highlighted with its corresponding nucleotide color.
