# Supplementary material for: Next-Generation Sequence Analysis of Cancer Xenograft Models
Source: PLoS One. 2013 Sep 26;8(9):e74432. doi: 10.1371/journal.pone.0074432 (PMC3784448; doi:10.1371/journal.pone.0074432)
Supplement: Table S1 — Summary of the total and post QA/QC number of sequenced reads from each NGS experiment performed. Control: peripheral blood BL209, Cell line: NCI-H209; Xenograft: xenograft sample derived from the NCI-H209 cell line. LX22, LX33 and LX33: SCLC primary xenograft lines LX22, LX33 and LX36. The number of mapped reads for the xenograft samples are human-specific only. PE: pair-ends. (PDF) [file pone.0074432.s005.pdf]

Table S1. Summary of the total and post QA/QC number of sequenced reads from each NGS experiment performed. Control: peripheral blood BL209, Cell line: NCI-H209; Xenograft: xenograft sample derived from the NCI-H209 cell line. LX22, LX33 and LX33: SCLC primary xenograft lines LX22, LX33 and LX36. The number of mapped reads for the xenograft samples are human-specific only. PE: pair-ends.

	Exon capture			Whole genome			RNA-Seq		
	Control	Cell line	Xenograft	Control	Cell line	Xenograft	LX22	LX33	LX36
Total reads (no)	58 10^{6}	66 10^{6}	82 10^{6}	66 10^{6}	75 10^{6}	78 10^{6}	54 10^{6}	86 10^{6}	89 10^{6}
Passed QA/QC reads (PE, no.)	52 10^{6}	59 10^{6}	74 10^{6}	55 10^{6}	65 10^{6}	68 10^{6}	47 10^{6}	78 10^{6}	80 10^{6}
