# Supplementary material for: Next-Generation Sequence Analysis of Cancer Xenograft Models
Source: PLoS One. 2013 Sep 26;8(9):e74432. doi: 10.1371/journal.pone.0074432 (PMC3784448; doi:10.1371/journal.pone.0074432)
Supplement: Table S2 — Summary of the alignments statistics of each NGS experiment performed. LX22, LX33 and LX33: SCLC primary xenograft lines LX22, LX33 and LX36.Control: peripheral blood BL209, Cell line: NCI-H209; Xenograft: xenograft sample derived from the NCI-H209 cell line. The number of mapped reads for the xenograft samples are human-specific only. For the exome capture and low-coverage whole genome analyses of the xenograft sample concordantly paired reads were analyzed. (PDF) [file pone.0074432.s006.pdf]

Table S2. Summary of the alignments statistics of each NGS experiment performed. LX22, LX33 and LX33: SCLC primary xenograft lines LX22, LX33 and LX36.Control: peripheral blood BL209, Cell line: NCI-H209; Xenograft: xenograft sample derived from the NCI-H209 cell line. The number of mapped reads for the xenograft samples are human-specific only. For the exon capture and low-coverage whole genome analyses of the xenograft sample concordantly paired reads were analyzed.

		Total reads mapped (no,%)	Pairs concordantly mapped (no, %)	Reads with Phred alignment score ≥ 20 (no, %)	Singletons (no., %)
RNA-Seq	LX22	80,519,547 (86.1)	74,968,964 (80.0)	75,945,281(94.3)	1,952,903 (2.0)
	LX33	136,808,432 (88.0)	130,742,872 (92.0)	127,872,221 (93.5)	2,518,180 (1.6)
	LX36	148,796,811(89.7)	137,261,948 (85.8)	135,272,944 (90.9)	2,566,927 (1.6)
Exon capture	Control	97,458,075 (94.2)	91,924,276 (88.8)	82,441,340 (84.6)	2,112,581 (2.0)
	Cell line	112,171,412 (94.6)	106,747,096 (90.0)	97,449,340 (86.9)	2,112,467 (1.8)
	Xenograft	128,604,306 (87.2)	124,892,360 (84.6)	110,874,569 (86.2)	(-.-)
Low-coverage whole genome	Control	104,535,862 (94.1)	102,613,686 (92.3)	99,832,086 (95.5)	778,308 (0.7)
	Cell line	123,505,627 (94.7)	121,364,648 (93.0)	118,273,136 (95.8)	828165 (0.6)
	Xenograft	96,493,162 (70.9)	94,676,296 (69.6)	92,869,442 (96.2)	362 (-.-)
