# Supplementary material for: Next-Generation Sequence Analysis of Cancer Xenograft Models
Source: PLoS One. 2013 Sep 26;8(9):e74432. doi: 10.1371/journal.pone.0074432 (PMC3784448; doi:10.1371/journal.pone.0074432)
Supplement: Table S3 — Summary of the depth of coverage obtained for the exome capture (A) and low pass whole genome (B) experiments. Control: peripheral blood BL209, Cell line: NCI-H209; Xenograft: xenograft sample derived from the NCI-H209 cell line. The results of the xenograft sample only represent those reads that were human specific. Depth of coverage was estimated on whole reads. (PDF) [file pone.0074432.s007.pdf]

Table S3. Summary of the depth of coverage obtained for the exon capture (A) and low pass whole genome (B) experiments. Control: peripheral blood BL209, Cell line: NCI-H209; Xenograft: xenograft sample derived from the NCI-H209 cell line. The results of the xenograft sample only represent those reads that were human specific. Depth of coverage was estimated on whole reads.

A

		Depth of coverage (mean)	Bases covered at least X times (%)					Duplication levels (%)
			1X	3 X	5 X	7 X	10 X	
Low pass whole genome	Control	3.5	94.4	63.6	26.3	8.8	1.1	0.4
	Cell line	4.2	95.1	70.7	39.3	16.8	3.3	0.4
	Xenograft	3.3	91.5	57.6	25.3	8.5	1.3	0.2

B

		Depth of coverage (mean)	Bases covered at least X times (%)					Duplication levels (%)
			15 X	30 X	50 X	75 X	100 X	
Exon capture	Control	107.2	93.9	83.2	66.9	49.1	35.6	10.0
	Cell line	108 .6	94.5	84.7	68.5	50.3	36.6	10.0
	Xenograft	136.1	95.2	87.4	74.7	59.3	46.4	15.4
