# Supplementary material for: Next-Generation Sequence Analysis of Cancer Xenograft Models
Source: PLoS One. 2013 Sep 26;8(9):e74432. doi: 10.1371/journal.pone.0074432 (PMC3784448; doi:10.1371/journal.pone.0074432)
Supplement: Table S4 — Table describing sample-specific single nucleotide variants. (A) Xenograft-specific non-synonymous coding variants. (B) Cell line specific non-synonymous coding variants. For sample-specific variants, approximate read depth is shown (reads with MQ = 255 or with bad mates were filtered). Read and allelic depth of the sample were the variant was not identified were calculated on de-duplicated reads. NSC: non-synonymous coding variant. (PDF) [file pone.0074432.s008.pdf]

Table S4. Table describing sample-specific single nucleotide variants. (A) Xenograft-specific non-synonymous coding variants. (B) Cell line specific non-synonymous coding variants. For sample-specific variants, approximate read depth is shown (reads with MQ=255 or with bad mates were filtered). Read and allelic depth of the sample were the variant was not identified were calculated on de-duplicated reads. NSC: non-synonymous coding variant.

A

						Xenograft Sample		Cell Line Sample (variant not called)	
Variant genomic position (chr:position)	Id.	Reference genome base	Alternative allele base	Gene modified	Predicted variant effect	Read depth (no.)	Allelic read depth (ref. : alt.)	Read depth (no.)	Allelic read depth (ref. : alt.)
chr1:6509142	.	T	C	ESPN	NSC-missense	10	6:4	-	-:-
chr1:14925635	.	C	T	KAZN	NSC-missense	11	3:8	8	8:-
chr1:14925653	.	G	A	KAZN	NSC-missense	8	2:6	5	5:-
chr3:49725034	rs142964215	T	A	APEH	NSC-missense	82	71:19	91	78:13
chr3:195453249	rs3828407	T	C	MUC20	NSC-missense	52	45:11	43	38:5
chr7:100677645	rs114262718	C	A	MUC17	NSC-missense	147	127:36	161	140:21
chr7:100679058	rs147856413	A	G	MUC17	NSC-missense	39	34:9	30	28:2
chr7:100679197	.	A	C	MUC17	NSC-missense	29	25:6	43	38:5
chr7:100679254	rs199808245	A	G	MUC17	NSC-missense	48	38:14	71	60:11
chr7:100683053	rs200821451	A	G	MUC17	NSC-missense	43	32:15	62	41:21
chr9:33385852	rs202183465	C	T	AQP7	NSC-missense	584	471:113	452	379:72
chr19:53058153	.	T	A	ZNF808	NSC-missense	34	28:8	36	36:-
chr19:53058157	.	A	G	ZNF808	NSC-missense	34	28:8	40	40:-
chr19:53058173	.	G	C	ZNF808	NSC-missense	35	30:7	45	45:-
chrX:114541228	.	A	G	LUZP4	NSC-missense	36	32:7	30	30:3

B

						Cell Line Sample		Xenograft Sample (variants not called)	
Variant genomic position (chr:position)	Id.	Reference genome base	Alternative allele base	Gene modified	Predicted variant effect	Read depth (no.)	Allelic read depth (ref. : alt.)	Read depth (no.)	Allelic read depth (ref. : alt.)
chr3:49413009	rs11552758	C	T	RHOA	NSC-missense	73	44:35	76	75,1
chr7:100684700	.	T	C	MUC17	NSC-missense	27	15:14	54	45,9
chr7:100684701	.	A	C	MUC17	NSC-missense	28	16:14	55	46,9
chr11:5717651	.	C	A	TRIM22	NSC-missense	108	95:23	263	260,3
chr11:67767049	.	G	T	UNC93B1	NSC-missense	60	52:14	135	135,-
chr11:95712127	.	C	A	MAML2	NSC-missense	80	65:23	93	92,1
chr14:62203741	.	A	C	HIF1A	NSC-missense	51	35:20	86	84,2
chr17:15449158	rs56222179	A	G	FAM18B2-CDRT4	NSC-missense	126	111:27	199	186,13
chrX:19086859	.	C	A	GPR64	NSC-missense	36	28:10	68	64,4
